# Design of Novel Phosphatidylinositol 3-Kinase Inhibitors for Non-Hodgkin’s Lymphoma: Molecular Docking, Molecular Dynamics, and Density Functional Theory Studies on Gold Nanoparticles

**DOI:** 10.3390/molecules28052289

**Published:** 2023-03-01

**Authors:** Abdalrahim M. Ali, Alaa A. Makki, Walaa Ibraheem, Mohammed Abdelrahman, Wadah Osman, Asmaa E. Sherif, Ahmed Ashour, Sabrin R. M. Ibrahim, Kholoud F. Ghazawi, Waad A. Samman, Abdulrahim A. Alzain

**Affiliations:** 1 Department of Pharmaceutical Chemistry, Faculty of Pharmacy, University of Gezira, Gezira 12217, Sudan; 2 Department of Pharmaceutics, Faculty of Pharmacy, University of Gezira, Gezira 12217, Sudan; 3 Department of Pharmacognosy, Faculty of Pharmacy, Prince Sattam Bin Abdulaziz University, Al-kharj 11942, Saudi Arabia; 4 Department of Pharmacognosy, Faculty of Pharmacy, University of Khartoum, Al-Qasr Ave, Khartoum 11111, Sudan; 5 Department of Pharmacognosy, Faculty of Pharmacy, Mansoura University, Mansoura 35516, Egypt; 6 Preparatory Year Program, Department of Chemistry, Batterjee Medical College, Jeddah 21442, Saudi Arabia; 7 Department of Pharmacognosy, Faculty of Pharmacy, Assiut University, Assiut 71526, Egypt; 8 Clinical Pharmacy Department, College of Pharmacy, Umm Al-Qura University, Makkah 24382, Saudi Arabia; 9 Department of Pharmacology and Toxicology, College of Pharmacy, Taibah University, Al-Madinah Al-Munawwarah 30078, Saudi Arabia

**Keywords:** cancer, pi3k, umbralisib analogues, molecular docking, molecular dynamics, gold nanoparticles, drug discovery, health and wellbeing

## Abstract

Non-Hodgkin’s lymphomas are a diverse collection of lymphoproliferative cancers that are much less predictable than Hodgkin’s lymphomas with a far greater tendency to metastasize to extranodal sites. A quarter of non-Hodgkin’s lymphoma cases develop at extranodal sites and the majority of them involve nodal and extranodal sites. The most common subtypes include follicular lymphoma, chronic/small lymphocytic leukaemia, mantel cell lymphoma, and marginal zone lymphoma. Umbralisib is one of the latest PI3Kδ inhibitors in clinical trials for several hematologic cancer indications. In this study, new umbralisib analogues were designed and docked to the active site of PI3Kδ, the main target of the phosphoinositol-3-kinase/Akt/mammalian target of the rapamycin pathway (PI3K/AKT/mTOR). This study resulted in eleven candidates, with strong binding to PI3Kδ with a docking score between −7.66 and −8.42 Kcal/mol. The docking analysis of ligand–receptor interactions between umbralisib analogues bound to PI3K showed that their interactions were mainly controlled by hydrophobic interactions and, to a lesser extent, by hydrogen bonding. In addition, the MM-GBSA binding free energy was calculated. Analogue 306 showed the highest free energy of binding with −52.22 Kcal/mol. To identify the structural changes and the complexes’ stability of proposed ligands, molecular dynamic simulation was used. Based on this research finding, the best-designed analogue, analogue 306, formed a stable ligand–protein complex. In addition, pharmacokinetics and toxicity analysis using the QikProp tool demonstrated that analogue 306 had good absorption, distribution, metabolism, and excretion properties. Additionally, it has a promising predicted profile in immune toxicity, carcinogenicity, and cytotoxicity. In addition, analogue 306 had stable interactions with gold nanoparticles that have been studied using density functional theory calculations. The best interaction with gold was observed at the oxygen atom number 5 with −29.42 Kcal/mol. Further in vitro and in vivo investigations are recommended to be carried out to verify the anticancer activity of this analogue.

## 1. Introduction

Non-Hodgkin’s lymphoma encloses the malignancies of the lymphoid nodes due to the clonal expansion of T cells, B cells, NK cells, or their precursor cells [1]. Malignancy can occur at each stage of their maturation and, depending on that, it is classified in most clinical trials as very aggressive or indolent lymphomas [2]. Indolent lymphomas contribute to more than a third of the cases of non-Hodgkin’s lymphoma [2]. Its subtypes include follicular lymphoma, chronic/small lymphocytic leukaemia, mantel cell lymphoma, and marginal zone lymphoma [3]. Pharmacotherapy typically started with alkylating agents such as cyclophosphamide, doxorubicin, and vincristine [4]. However, advanced newer therapies have emerged such as monoclonal antibodies and small molecules targeted therapies which specifically inhibit pathways that are essential for cancer cells’ survival and growth [4]. For example, proteasome inhibitors, histone deacetylase inhibitors, Bruton’s tyrosine kinase inhibitors, nuclear export inhibitors, and phosphatidylinositol 3-kinase inhibitors have been used [2,3,4]. The phosphatidylinositol 3-kinase (PI3Ks) is a group of lipid kinases that phosphorylate phosphatidylinositols at the 3’ position of the inositol ring after activation by G protein-coupled receptors and tyrosine kinase receptors [5]. They are classified into several classes. Regulatory and catalytic subunits make up the heterodimeric Class I PI3K enzymes [6]. They are further divided according to sequence similarity into IA and IB subsets. The substantially homologous class IA isoforms include PI3Kα, PI3Kβ, PI3Kγ, and PI3Kδ, each with distinct non-redundant biological functions [7]. Moreover, these functions are related to the capability of class IA PI3Ks to activate protein kinase B in the PI3K/AKT/mTOR pathway, which is involved in signalling for increased cell growth, metabolism, and cell-cycle progression with oncogene phosphatidylinositol-4,5-bisphosphate-3-kinase (PIK3CA) and tumour phosphatase tensin homologue (PTEN) mutations [7,8]. The latter is thought to play an important role in how responsive malignant tumours are to insulin, IGF1, and calorie restriction [9].

Isoform selectivity has been a key consideration in driving inhibitor design and development. Accordingly, the PI3Kδ isoform that has a largely restricted expression to the hematopoietic system with an important role in regulating the immune system; thus, it became a frequent target in drug discovery and development [10,11].

Idelalisib, aPI3Kδ inhibitor, can effectively induce chronic lymphocytic lymphoma (CLL) cells for apoptosis in culture. It was authorised for the treatment of CLL, relapsed follicular B-cell non-Hodgkin lymphoma, and relapsed small lymphocytic lymphoma by the US FDA in July 2014 [9,12]. A second-generation PI3K inhibitor is duvelisib. The US FDA authorised its use in adult patients with relapsed or refractory CLL, small lymphocytic lymphoma, or relapsed or refractory FL [9,12]. 

Umbralisib (also known as TGR-1202) is one of the latest orally bioavailable PI3K inhibitors that has been found to function, at sufficient concentrations, as a casein kinase 1 epsilon (CK1ε) inhibitor [13]. Umbralisib exhibited improved selectivity for PI3Kδ compared with other PI3Kδ inhibitors. Trails demonstrated the potential of umbralisib as monotherapy or as a part of dual or triple combination therapies [14]. In addition, comparing umbralisib to the authorised PI3Kδ inhibitors idelalisib and duvelisib has repeatedly shown that umbralisib has reduced occurrences of immune toxicities [15].

Usually, the process of drug discovery can take more than a decade, with huge expenditure in terms of cost and time. However, the use of computational techniques can considerably reduce the time and cost in comparison to traditional experimental methods [16]. The discovery of saquinavir is an instance of the success of computational methods in drug discovery [17]. During recent years, a lot of research emerged using computational methods in drug analogues design, for instance, the in silico design and evaluation of 5-fluorouracil analogues as potential anticancer agents [18].

Traditional cancer treatments may result in inadequate cancer eradication or the destruction of healthy cells [19]. Nanotechnology addresses the problem by providing tailored chemotherapies extending efficacy, selectivity, and reducing toxicity [20]. If employed as a drug carrier, the nanoparticle drug system should guide to the site of action without alteration and function in a chemically proper manner to combat illness at the chosen target. Understanding the sort of association seen between the nanoparticle and the drug is therefore critical for therapeutically optimum drug carrier design [21].

In this research, several computational methods have been used to design new umbralisib analogues targeting the PI3Kδ protein for non-Hodgkin’s lymphoma.

## 2. Materials and Methods

Maestro v 12.8 of the Schrodinger suite was utilised for the in silico studies. DFT calculations were performed by Gaussian 09.

### 2.1. Protein Preparation

The crystal structure of the phosphoinositide 3-kinase delta complex with idelalisib (PDB ID:4XE0) was obtained from the protein data bank [22] and the protein preparation wizard tool was used to prepare the protein. The protein structure was pre-processed by removing all solvent molecules beyond 5 Å from het groups, assigning bond orders, and adding all hydrogens. Prime was employed to add missing side chains and loops, and Epik was used to generate hit states. Then, the pre-processed structure was optimised and minimised with the OPLS4 force field [23].

### 2.2. Receptor Grid Generation

Grid generation was carried out using the grid generation wizard using co-crystallised idelalisib to define the receptor [24]. In the receptor grid generation with a partial atomic charge of 0.25, the scaling factor for the protein van der Waals radii was set to 1.0 to soften the potential for nonpolar regions of the receptor where no constraints were applied. The grid box was constructed at the centroid of the co-crystallised ligand by a radius of 10 Å around the crystal structure’s ligand. The default grid size was adopted from the Glide program.

### 2.3. Quantum Polarised Ligand Docking (QPLD)

Due to the lack of a crystal structure of umbralisib bound to PI3Kδ up to now, the three-dimensional structure of umbralisib was retrieved from PubChem and optimised by Jaguar through the DFT (B3LYP) functional and 6-31G** base set. Using the Glide module, QPLD was performed instead of molecular docking. QPLD describes the interactions implicated in molecular associations of ligands to the protein active site using quantum mechanics, hence ensuring a better accuracy compared to molecular docking that relies on molecular mechanics [25]. We utilised the same grid file for QPLD that was created by the Glide grid-generating tool. The initial docking was carried out by Glide using the extra-precision mode, followed by re-docking using the extra-precision docking mode. The quantum charges were calculated by QSite through a single-point energy calculation on each complex, with the 6-31G*/LACVP* basis set, the B3LYP density functional, and the “Ultrafine” SCF accuracy level (iacc = 1, iacscf = 2). Finally, selection was completed using the Glide score [24]. The native co-crystalised ligand, idelalisib, was used as the reference for docking, and it poses validation through RMSD calculations.

### 2.4. Analogues Design

The best re-docked pose generated from the QPLD of umbralisib into the binding pocket of 4XE0 was used as the input for the Ligand Designer tool [26]. The analogues were designed through the visualisation of umbralisib that is docked into the binding pocket of 4XE0. The drug’s surroundings were examined, including the growth space between the ligand and the protein, and the locations of the protein potential interaction sites such as hydrogen bond donors, acceptors, rings, and hydrophobic groups permitted the modification of the drug to increase its binding to the protein. Isostere scanning was performed with amine, amide, halides, methyl, and hydroxyl (Cl, F, Br). A library of 2898 fragments was used for the enumeration to form new interactions and fill the growth space in the receptor. This produced analogues which were automatically docked to the protein, and any structures that did not achieve a good pose were automatically removed [27].

### 2.5. Extra Precision Docking

The designed ligands were prepared using LigPreb, with Epik for energy minimisation, ionisation, and tautomerisation. The ligands were docked with the active site utilizing ‘extra precision’ glide docking (Glide XP), which allows the ligands to attach flexibly. Glide produces conformations internally, which are then sent via several filters. Only active compounds will have accessible poses in XP docking that avoid these penalties while also receiving good ratings for a suitable hydrophobic contact between the protein and the ligand, hydrogen–bonding interactions, and other factors. The XP method’s goals are to eliminate false positives and improve the link between exceptional poses and good results [28]. A Glidescore function was used to choose the best-docked structure. Glidescore is a modified and expanded version of the empirical base function [29]. The favourable conformations of ligands in the active site were searched and the results were compared against umbralisib .The docking ligand conformations were validated by the RMSD calculations against the native co-crystalised ligand idelalisib [30].

### 2.6. Determination of ADMET Properties

The ADMET properties of the docked ligands were calculated by the QikProp module of Maestro. Lipinski’s rule of five and various descriptors were computed to determine the drugability and safety of the designed analogues [31]. Additionally, the potential toxicity of the designed analogues was predicted using the ProTox-II web server [32]. It was used to predict the possible hepatic toxicity, carcinogenicity, immune toxicity, mutagenicity, and cytotoxicity.

### 2.7. MM-GBSA Calculations

The molecular mechanics energies with generalised born and surface area (MM-GBSA) free energy binding of the top ligand–protein complexes were evaluated by the MM-GBSA continuum solvation method using the Prime module of Maestro using the OPLS4 force field and VSGB solvation model [33].

### 2.8. Molecular Dynamics (MD)

The MD simulation of the selected top umbralisib designed analogue was performed using the Desmond software. The Glide XP docking output files were used as the input. The system was neutralised by the addition of sodium and chloride ions and a simulated triclinic periodic boundary box with an extension of 10 Å from each direction was created. The solvent model (TIP3P, the transferable intermolecular potential 3 points) was employed for each system. Energy minimisation was applied to the systems until a gradient threshold of 25 Kcal/mol was attained at 300 K and 1 bar pressure using the NPT ensemble class. Each system was subjected to separate MD runs for 100 ns via an NPT ensemble. The particle mesh Ewald (PME) algorithm was adopted to calculate long-range Coulombic contacts, whereas the RESPA integrator was employed to govern any covalent bonds which are linked with hydrogen atoms. The inner time step was 2 fs all across the simulation. Regarding short-range electrostatic interactions, a cut-off value of 9.0 was used; however, for long-range Van der Waals (VDW) interactions, a uniform density approximation was employed. The Nosé–Hoover thermostat was employed at a 300 K temperature and 1 atmospheric pressure, whereas the Martyna–Tobias–Klein barostat was applied to conserve the condition throughout the simulation. Next, based on the MD simulation’s trajectory data, the stability of each system was assessed by using RMSD (root mean square deviation), RMSF (root mean square fluctuation), and ligand–protein contacts [33].

### 2.9. DFT and Gold Nanoparticles

Gaussian 09 was used to carry out the density functional theory calculations. The B3LYP functional and LANL2DZ basis set were used for optimisation and frequency calculations in a vacuum. A gold cluster of 4 atoms [19] with an approximately diameter of 5 Å and a height of 3 Å was used. The gold and the top compound were each optimised individually. Then, to simulate interactions between them, ten complexes of the nanocluster and the top compound were generated and optimised to locate the best site for interactions. The equation below was used to calculate the interaction energy [21].

Interaction energy = complex energy − gold energy − compound energy

## 3. Results

### 3.1. Quantum Polarised Ligand Docking (QPLD) and Analogues Design

The initial charges were computed and used to re-dock the ligand in the active site with a higher accuracy. Table 1 presents the best ten poses generated through the QPLD algorithm. The docking scores ranged from −7.49 to −6.74 Kcal. The resulting docking pose’s divergence from the matching co-crystallised pose of the identical ligand molecule was measured using the root mean square deviation (RMSD). The docking protocol was validated by the co-crystalised ligand idelalisib. The RMSD was within the acceptable range of less than 2 Å [34]. QPLD uses force field components and the XP docking score to rank the docking poses. Pose one showed the best score and rank among all those generated. 

**Figure 1:**
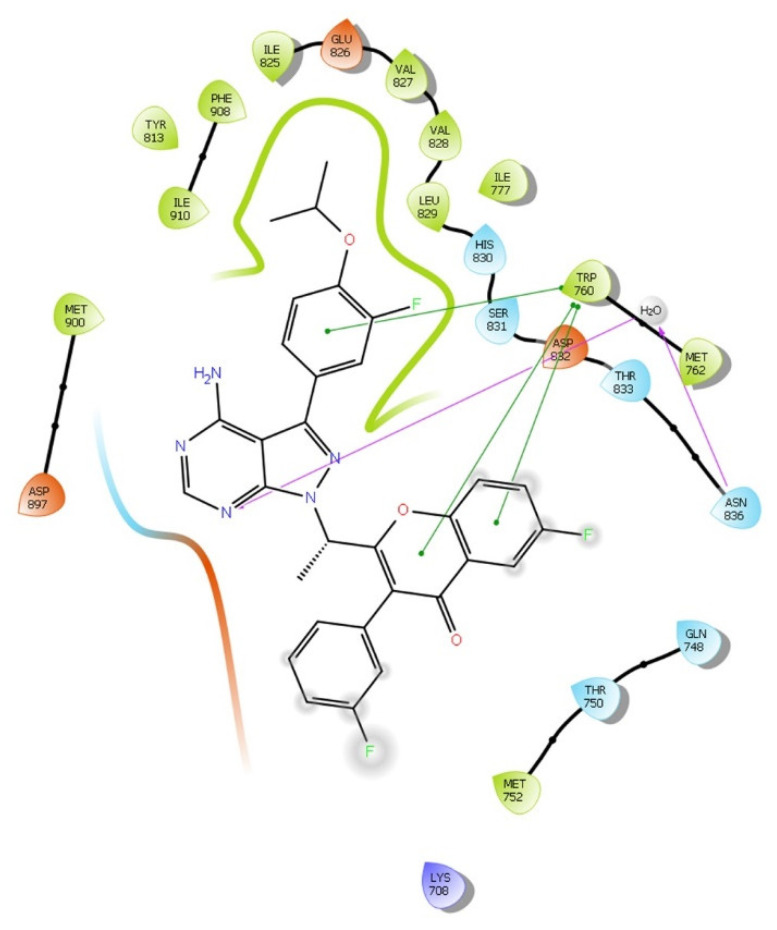
2D interaction of umbralisib in the active site of PI3Kδ protein (PDB ID:4XE0) using the QPLD tool of Maestro software. The hydrogen bond interactions with residues are represented by a purple dashed arrow directed towards the electron donor. The π-π interactions are represented by a green line.

In pose one, umbralisib formed two hydrophobic interactions with TRP760: one water-mediated hydrogen bond with ASN836, and the other non-bonding interactions are depicted in Figure 1.

**Figure 2:**
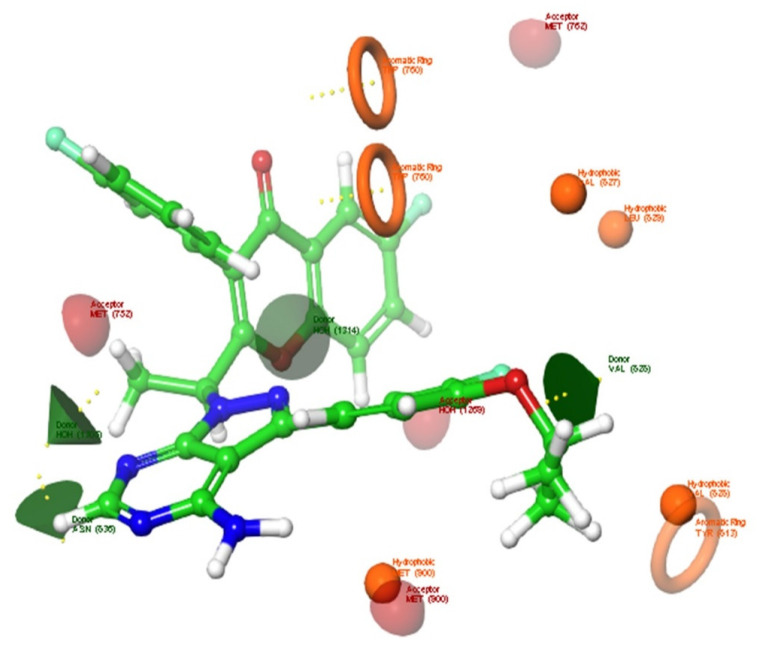
Potential interaction sites of umbralisib in the active site of PI3Kδ using the Ligand Designer tool of Maestro software. Potential hydrogen bond donor sites are indicated by green cones. While potential hydrogen bond acceptor interaction sites are indicated by red hemispheres. Potential hydrophobic interaction sites are indicated by yellow spheres and rings, respectively.

Pose one was used as the input for the Ligand Designer tool in the Schrodinger suite. An analysis of the complex and subsequent determination of the potential interaction sites was performed. As shown in Figure 2, the hydrogen bond donor residues were ASN836 and VAL828, while the hydrogen bond acceptor residues were MET762, MET752, MET900, and GLN748. The hydrophobic residues VAL827, VAL828, LEU829, TRY813, MET900, and TRP760 were noticed. Then, enumerations were performed using the Ligand Designer tool fragments library to optimize umbralisib binding to the protein by forming new interactions and extending the existing ones. This generated 391 umbralisib analogues.

### 3.2. Extra Precision Docking and MM-GBSA Calculations

**Figure 3:**
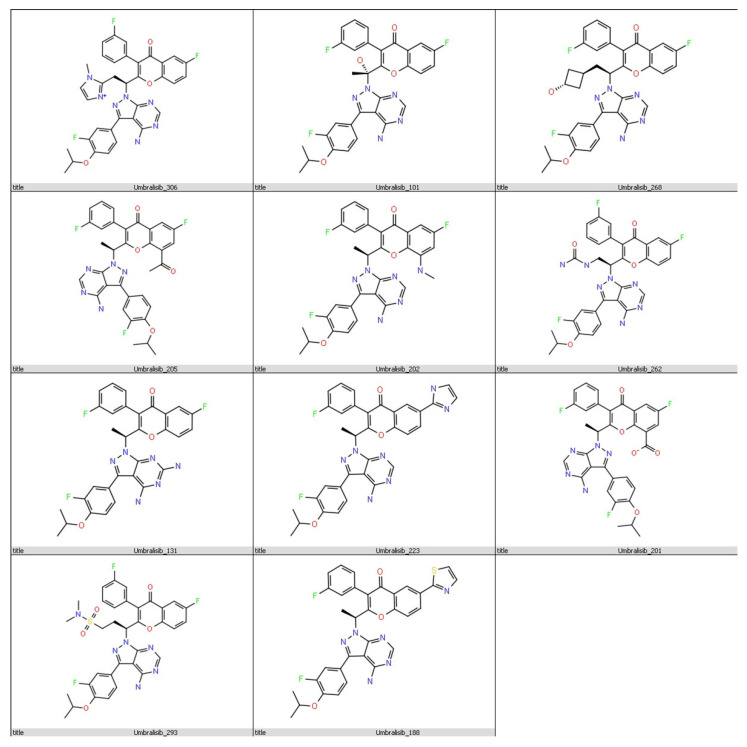
Chemical structures of the eleven analogues that showed promising binding affinity against PI3Kδ protein (PDB ID: 4XE0) using the Ligand Designer tool of Maestro software.

An extra precision (XP) docking mode was performed to determine the affinity of the designed analogues towards PI3Kδ. Among the 391 designed analogues listed in the Supplementary Materials with their structures (Table S1), 376 analogues had a favourable binding to PI3Kδ with negative docking scores between −3.75 and −8.46. Eleven analogues (Table 2 and Figure 3) had a docking score from −7.632 to −8.425 Kcal/mol. Therefore, they have a better affinity towards PI3Kδ compared to umbralisib, which only had a docking score of −7.58 Kcal/mol. The best eleven analogue docking conformations were all within the acceptable RMSD limit (less than 2 Å).

**Figure 4:**
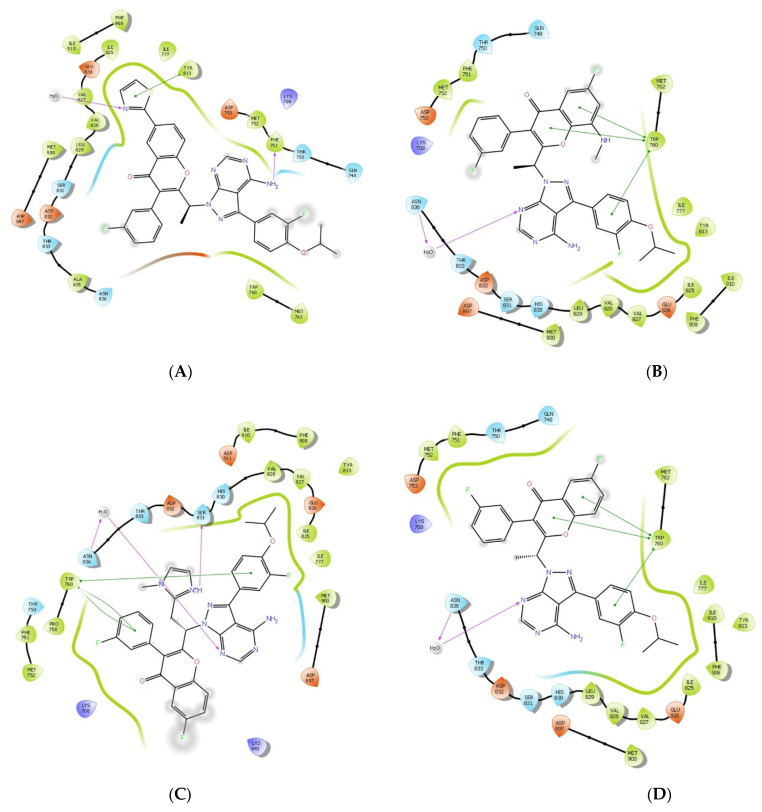
2D interaction of the top three analogues and the reference in complex with PI3Kδ protein (PDB ID:4XE0) using the XP docking mode of Glide software. The hydrogen bond interactions with residues are represented by a purple dashed arrow directed towards the electron donor. The hydrophobic residues are in green colour. (**A**) Analogue 188, (**B**) analogue 202, (**C**) analogue 306, and (**D**) umbralisib.

**Figure 5:**
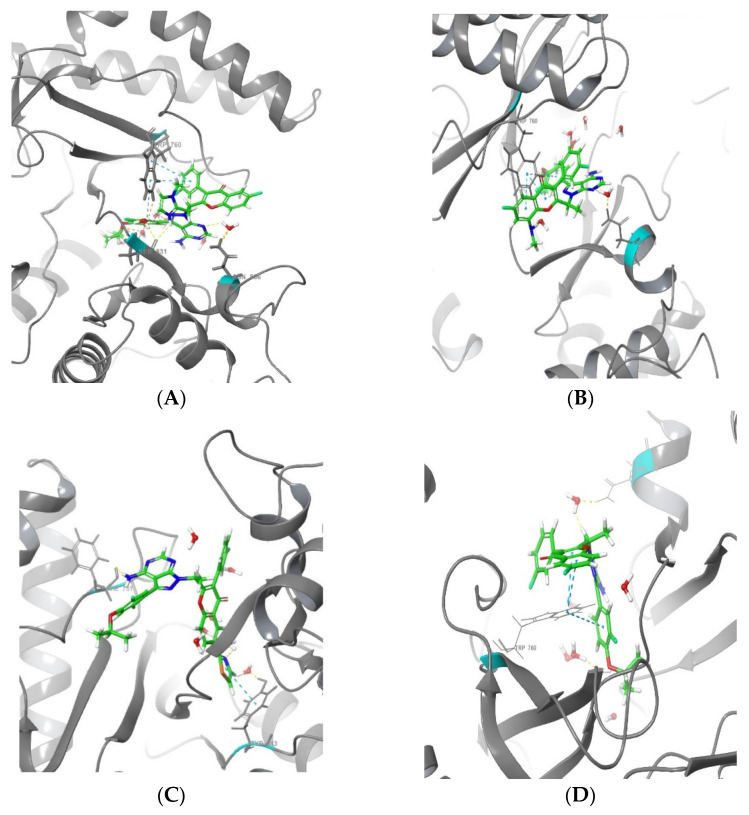
Binding poses of (**A**) Analogue 306 PI3Kδ complex. (**B**) Analogue 202 PI3Kδ complex. (**C**) Analogue 188 PI3Kδ complex. (**D**) Umbralisib PI3Kδ complex. Ligands are shown in the stick models. PI3Kδ is shown in the ribbon model (blue dashed lines represent hydrophobic bonds, whereas yellow lines represent hydrogen bonds).

In Table 2, the top eleven analogues exhibited interactions with the amino acid residues TRP760, ASN836, HIS830, PHE751, VAL828, SER831, and TRY813 in the binding pocket of PI3Kδ. The analysis of the interaction patterns showed that the top eleven designed analogues were stabilised in the PI3Kδ active site by both hydrogen bonds and hydrophobic interactions. The designed analogues barely exhibited any electrostatic or other sorts of interaction. Almost all the hydrophobic interactions of the designed analogues were formed with TRP760 and TRY813. The amino acid residues ASN836, HIS830, PHE751, ASP911, VAL828, and SER831 showed hydrogen bond interactions with the designed analogues. The interactions of the best three analogues, 188, 202, and 306, are shown in Figure 4 and Figure 5, respectively. 

Analogue 188 formed one hydrogen bond with the residue of PHE751 at 2.49 Å, as a donor with the amino group. One hydrophobic interaction occurred with the residue of TRY813 at 4.48 Å, while analogue 202 formed one water-mediated hydrogen bond at residue ASN836 as an acceptor with the nitrogen atom at a 4.2 Å distance. Besides that, three Pi-Pi interactions with TRP760 were observed at 5.37, 3.94, and 4.13 Å. Analogue 306 interacted by one mediated hydrogen bond with residue ASN836 at 3.87 Å as an acceptor with the nitrogen atom, and another hydrogen bond with residue SER831 at 2.29 Å as a donor with the NH^+^ of the added methyl imidazole ring. In addition, it has formed three Pi-Pi interactions with TRP760 at 4.86, 5.34, and 5.25 Å. Umbralisib formed one water-mediated hydrogen bond with ASN836 at 3.95 Å, as an acceptor with the nitrogen atom, and three hydrophobic Pi-Pi interactions with TRP760 at 5.25, 4.0, and 4.13 Å. The interactions of the remaining eight analogues are shown in the Supplementary Materials (Figure S1).

**Figure 6:**
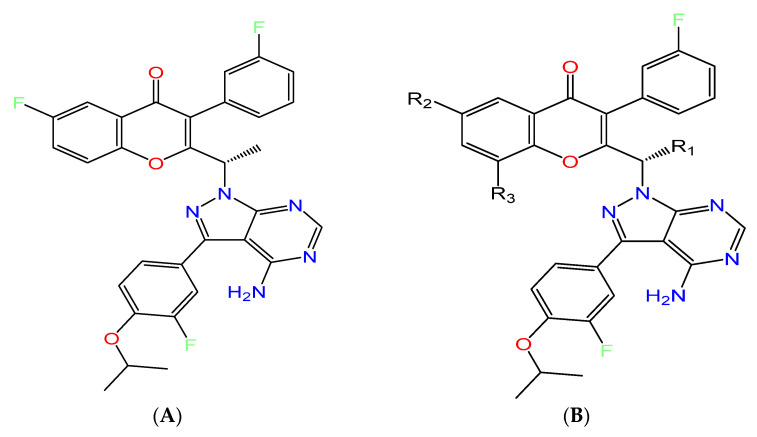
(**A**) Structure of umbralisib. (**B**) The substitution pattern observed in the best-designed analogues.

The top eleven analogues were further subjected to MM-GBSA to determine the binding free energy in solvent, thus providing more accurate results. The top eleven analogues displayed MM-GBSA results from −44.59 to −52.21 Kcal/mol, whereas umbralisib’s MM/GBSA was only −44.43 Kcal/mol (Table 2). All the top eleven designed analogues show the addition of a hydrogen bond-forming substituent at one of three positions, R_1_, R_2_, or R_3_, as in Figure 6.

The addition of a small polar group at R_3_ has led to enhanced docking scores. Heterocyclic aromatic or hydrogen bond donor moieties at R_2_ as well as hydrogen bond acceptors at R_3_ have also improved the binding affinity. The replacement of the iso-propoxy group cannot be tolerated. The replacement of fluorine with other halogens, such as chlorine, bromine, and iodine substituent, also has considerably reduced the binding affinity. However, an extended substitution in R_1_ was critical for the overall improvement in binding for the majority analogues.

**Figure 7:**
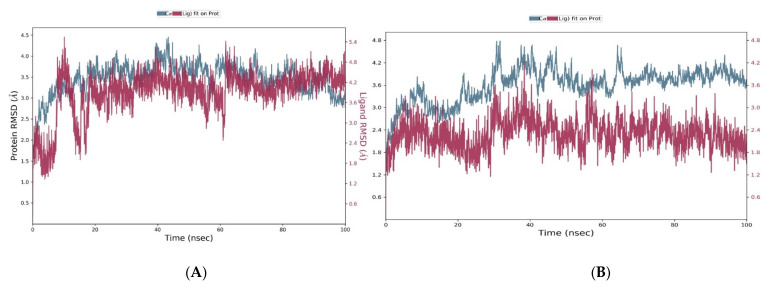
The protein–ligand RMSD plot of the top compound and the reference complexed with PI3Kδ protein (PDB ID:4XE0) during 100 ns molecular dynamics simulation using Desmond software. (**A**) Analogue 306 and (**B**) umbralisib.

There are distinct regions within the binding site of PI3K (p110) that are frequently targeted in the design of isoform-selective PI3K inhibitors: the hinge region, region Ⅰ, region Ⅱ, specificity pocket, affinity pocket, and Tryptophan self [7]. The hinge region is fairly conserved among all PI3K isoforms. In umbralisib and analogues 202, 306, and 188, the amino-pyrazole pyrimidine heterocyclic system serves as the hinge-binding moiety [7]. Region 1 non-conserved residues (also known as the ribose-binding region or hydrophobic region II) include the non-conserved residues in region I, including δAsp832 and δAsn836. Umbralisib and analogues 202, 306, and 188 formed hydrogen bonds with ASN863, as seen in Figure 4. The tryptophan shelf is generated by the non-conserved Thr750, which permits inhibitors to access the face of the conserved Trp760, which produces the tryptophan shelf [35]. Umbralisib and analogues 202, 188, and 306 have been found to target the tryptophan self, which is evident by the stable interactions formed with TRY760 (Figure 4). The specificity pocket was occupied by the chromone heterocyclic system in umbralisib and the analogues. Besides that, the affinity pocket was occupied by the fluro-iso-propoxy benzene moiety, as seen in Figure 7.

### 3.3. ADMET Analysis

Whereas many potential drug candidates exhibit efficacy in early investigations, they fall short because they lack the required pharmacokinetic characteristics. As a result, it is essential to calculate certain computational and predictable parameters. The selected eleven analogues were evaluated for their drug-likeliness properties with the help of the Lipinski rule of five. According to Lipinski’s rule of five, molecules with a molecular weight (MW < 500), hydrogen bond acceptor (HBA ≤ 10), hydrogen bond donor (HBD ≤ 5), and predicted octanol/water partition coefficient (QPlogPo/w < 5) are considered druggable candidates. In Table 3, analogue 262 violated one rule (MW < 500) as it has a molecular mass of 692.59. Other than that, the remaining analogues, alongside umbralisib, violated two rules (MW < 500 and QPlogP o/w < 5), with a molecular mass from 651.64 to 586.57 g/mol and QPlogPo/w values between 5.73 and 7.35. This indicated their hydrophobic nature, whereas in analogue 262, the presence of the amide substituent supports its low QPlogPo/w value of 4.596.

Furthermore, other ADMET parameters are reported in Table 3. Solubility, expressed as QPlogS, is a key property of a druggable molecule. Its optimum value varies from −2.0 to 6.5. All analogues fall above range, indicating their less soluble nature. The maximum solubility is shown by analogue 262 (−7.05) and the least solubility by analogue 188 (−10.02). When a drug is taken orally, it is absorbed in the gut and eventually reaches its designated target. All eleven analogues have demonstrated moderate-to-high human intestinal absorption.

The parameters, such as the blood–brain barrier factor (QPlogBB) and cellular membrane access factor (QPPCaco), have been studied to assess the permeability of the membranes. These values indicated good cellular access for all molecules with an inability to cross the blood–brain barrier. Considering the cardiotoxicity parameter (QPlogHERG), all analogues except analogue 201 (−4.82) showed slightly higher predicted IC_50_ values for HERG K^+^ channels blockage from −5.87 to −7.93.

### 3.4. In Silico Toxicity Prediction

Toxicology testing is an important step in the drug manufacturing and discovery phase, but it also requires additional financial investment. Various virtual tools have been developed for early testing and the eventual elimination of the substance from the drug race. Based on known databases, they can gain insight into the toxicity of test compounds, minimizing time, cost, and the need for animal testing [36]. In this scope, the ProTox-II virtual lab [32] was employed to predict the toxicity of umbralisib and the eleven analogues listed in Figure 3. In ProTox-II, oral toxicity is represented as the lethal dose (LD), at 50% (LD50) milligrams per kilogram weight of the test population. Umbralisib and all the eleven analogues are in class IV, with predicted lethal doses of 1000 mg/kg. This demonstrated their safety if ingested. Regarding liver toxicity, it can be observed that all eleven analogues and umbralisib do exhibit predicted hepatotoxicity, but umbralisib showed a higher probability for hepatoxicity, with a score of 0.64, than analogues 306, 101, and 268 that have only a probability score of 0.55. This showed a better safety profile than umbralisib in terms of hepatotoxicity. In terms of mutagenicity, analogues 306, 268, 262, 101, 131, and 293 are predicted to be free from mutagenicity, unlike umbralisib which showed active probability with a score of 0.51, respectively. Further, it was found that all analogues and umbralisib did not exhibit predicted cytotoxicity. Only analogue 293 showed predicted carcinogenicity with a score of 0.50. All analogues except analogue 188 exhibited predicted immunotoxicity, which is a favourable attribute in PI3K inhibitors as they target immune cells. Analogues 306, 268, 205, 202, 101, 131, and 202 exhibited a high probability of immunogenicity above 0.90, which corresponds with a stronger inhibitory activity than umbralisib against cancerous lymphocytes. 

### 3.5. Molecular Dynamics (MD)

Based on MM/GBSA and AMDET, analogue 306 had the best MM-GBSA binding free energy (−52.22) and the highest per cent human oral absorption (94%); therefore, it was selected for MD. The simulations were performed at 100 ns. To determine whether the protein–ligand complex is stable, the root mean square deviation (RMSD) of the C atoms was calculated. Furthermore, the root mean square fluctuation (RMSF) analysis and ligand–protein interactions were computed at numerous intervals during the simulation experiment.

In Figure 7A, the RMSD plot for the analogue 306-4XE0 system displayed that the protein had fluctuations at the initial 20 ns of the simulation, but later attained an equilibrium state of around 3.5 Å for the remaining 80 ns. MD simulation was also performed for the reference drug umbralisib. Figure 7B presents the RMSD plot for the umbralisib-4XE0 system. First, the protein displayed a high fluctuation for the first 60 ns of the simulation and equilibrated around 3.8 Å for the remainder of the simulation. The average of analogue 306 and the umbralisib ligand RMSD were 3 and 3.1 Å, respectively. Therefore, based on the RMSD profiles, we found that analogue 306 was more tightly bound to the protein than umbralisib was for most of the simulation. The analysis of the root mean square fluctuation (RMSF) provides the complex changes with time against each atom. 

**Figure 8:**
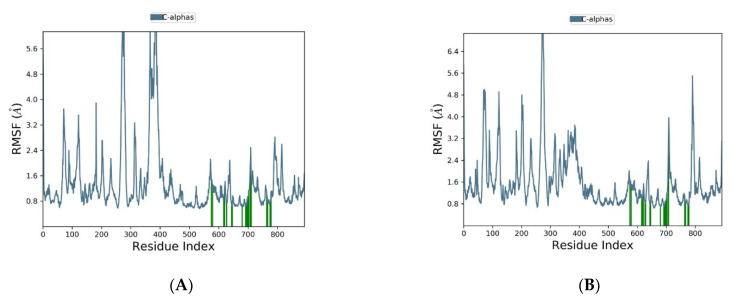
The RMSF plot of the PI3Kδ protein (PDB ID:4XE0) complexed the top compound and the reference during 100 ns molecular dynamics simulation using Desmond software. (**A**) Analogue 306 and (**B**) umbralisib.

Figure 8A,B show the protein RMSF of analogue 306 and umbralisib, respectively. The protein RMSF of analogue 306 displayed fluctuations in a range of 0.8 to 3.3 Å, except for the spikes values that were observed between residues 403 and 409 and 494 and 528. At the same time, the protein RMSF of umbralisib displayed fluctuations from 0.8 to 4.2 Å, except for the spike between residues 403 and 409. These designated residues in the two complexes might be in regions with extra-conformational flexibility. Furthermore, due to low residue mobility during the simulation period, the entire protein structure remained quite compact in both complexes. Even though the protein RMSF pattern was stable in both analogue 306 and umbralisib, the analogue 306 complex showed more reduced residues movement compared to the umbralisib complex. This finding demonstrated that analogue 306 produced a much more stable complex than umbralisib.

**Figure 9:**
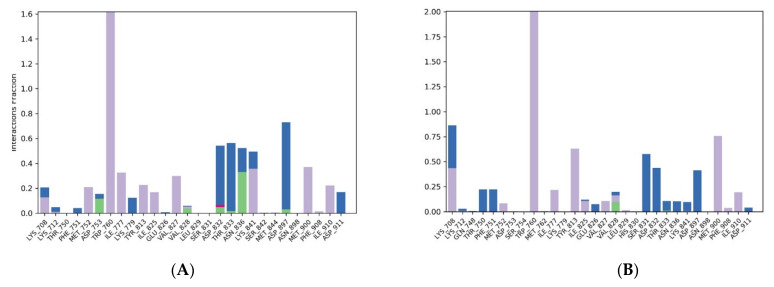
The protein–ligand contact histogram of the top compound and the reference complexed with PI3Kδ protein (PDB ID:4XE0) during 100 ns molecular dynamics simulation using Desmond software. (**A**) Analogue 306 and (**B**) umbralisib.

The interaction analysis showed that the interactions responsible for maintaining the stability in analogue 306 were direct and bridged hydrogen bond interactions mainly with ASP 897 (69%), THR833 (54%), and ASP832 (47%), and hydrophobic interactions with TRP760 (161%), as shown in Figure 9. The reference showed interactions with LYS708 (42%), SER831 (57%), ASP832 (43%), and ASP879 (41%) through direct and bridged hydrogen bonds as well as hydrophobic interactions with LYS708 (43%), TRP760 (180%), TRY813 (63%), and MET900 (75%).

### 3.6. DFT Calculations on Gold Nanoparticles

In order to determine the suitability of analogue 306 for delivery by gold nanoparticles, DFT calculations were performed. The interaction between the gold nanocluster and analogue 306 was studied at all three oxygen and seven nitrogen atoms of analogue 306 in the ten complexes listed in Table 4.

Analogue 306 and all ten complexes were examined by frequency calculations to obtain zero imaginary frequency. Zero point corrected electronic energy (EE + ZPE) was calculated to determine the energy of the gold nanocluster, analogue 306, and their complexes in the lowest vibrational state at absolute zero. The EE + ZPE was found to be −341, 342.8, and −1,417,316.68 Kcal/mol for each of the gold nanoclusters and analogue 306, respectively. In Table 4, the EE + ZPE was calculated for each complex. The interaction energy was calculated and found to be from −29.422 to −21.967 Kcal/mol. All the interaction energies listed in Table 5 were negative, indicating that the interaction between analogue 306 and gold is exothermic and suitable.

**Figure 10:**
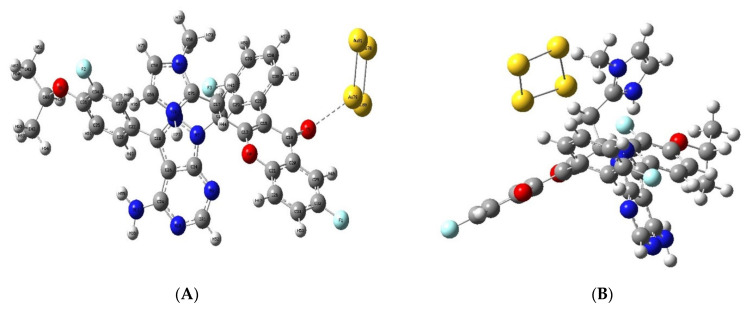
(**A**) Initial and (**B**) optimised geometry for the analogue 306 (oxygen atom number five) interaction with the gold atoms using Gaussian software.

The oxygen (No. 5) of analogue 306 (Figure 10) was found to be the most stable site to interact with gold at an interaction energy of −29.422 Kcal/mol, while the other complexes were less stable by 4 to 8 Kcal/mol.

## 4. Discussion

Despite considerable breakthroughs in the treatment of non-lymphoma Hodgkin’s (NHL), and the fact that some patients might be successfully managed and treated with a combination of immune-chemotherapy, subjects with relapsed and refractory lymphoma usually lose the fight against the disease [2]. New treatment targets are emerging as we obtain a better understanding of lymphoma biology and molecular aetiology [37]. Preclinical and clinical research on the inhibitors of phosphatidylinositol 3-kinase, AKT, the mammalian target of rapamycin, has established the importance of this pathway [38]. Umbralisib is one of the latest discovered PI3Kδ inhibitors [39]. Clinical trials are currently in the recruitment process and five are active for other indications; chronic lymphocytic leukaemia, small lymphocytic lymphoma, mantle cell lymphoma, and B-cell non-Hodgkin’s lymphoma, in combination with other small molecule anticancer drugs and biologicals [40].

Drug modification is an alternative approach for discovering superior molecules with a significantly increased selectivity, potency, and lower toxicity than the existing drugs [41]. Recently, it has been noted that drug alterations, such as the addition of halogen, alkyl, alkoxy, hydroxyl, and other groups, play a major role in improving drug performance [42]. Drug optimisation using in silico strategies is frequently studied in the literature; as an example, the rational design of 14 novel idelalisib derivatives (which is a drug that selectively inhibits PI3Kδ)k through QSAR, molecular docking, and molecular dynamic simulations [43].

In this research, we aimed to design umbralisib analogues through attempts to enhance the binding affinity towards PI3Kδ. Several computational methods have been used in this process to select the best alternatives. First, QPLD was carried out to generate the umbralisib posed in the PI3Kδ active site. Next, the ligand designer module was used to generate umbralisib analogues. Molecular docking produces and rates receptor–ligand poses depending upon their binding affinities. The designed analogues were evaluated by XP docking. The top eleven designed analogues had a docking score of <−7.58 Kcal/mol. These analogues showed hydrogen bonding interactions with residues ASN836, HIS830, PHE751, ASP911, VAL828, and SER831, while hydrophobic interactions were observed with residues TRP760 and TRY813. Experimental and computational research has produced comparable findings with various compounds. In a previous study conducted by Liu et al, in the PI3Kδ active site, amino acid residues VAL828, SER831, and ASN836 showed hydrogen bonds with the ligands, while residues TRP760 and TRY813 showed hydrophobic interactions with the ligands [8]. In another study, GSK pharmaceuticals developed a novel PI3Kδ inhibitor, nemiralisib, which showed hydrogen bonding with residues VAL828, TRY813, and ASP911 and hydrophobic interactions with the residue TRP760 [7]. The docking results were additionally confirmed by determining the free binding energy by the MM-GBSA method. MM-GBSA calculations suggested that our chosen analogues had a more favourable binding free energy than umbralisib.

Pharmacokinetic and toxicological features provide critical information on how drug molecules behave inside the human body. In this research, the drug-likeness and ADMET properties were calculated using QikProp. As reported in Table 3, most of the eleven analogues and umbralisib showed the same drug-likeness calculated by Lipinski’s rule of five. In addition, QikProp predicted that the eleven analogues would show a good absorption and distribution across the body. Regarding cell permeability, it is predicted that they will not cross the BBB. Thus, no central nervous system activity will be expected. Moreover, analogue 306 demonstrated a better safety profile with less predicted toxicity than umbralisib. 

Despite the excellent results of the previous investigations, which supported the potential of analogue 306 as a hit candidate, further confirmation and validation were obtained by molecular dynamics analyses. We did two dynamic simulations for analogues 306 and umbralisib. When analysing the structural stability using molecular dynamics simulations, the RMSD is a well-known metric. According to the RMSD interpretation of molecular dynamic simulations, the analogue 306 complex with the target is much more stable than the umbralisib complex, based on the low RMSD values recorded during the entire simulation. Furthermore, the minimal changes in the RMSF values suggested that the analogue 306 complex was structurally the most stable.

Regardless of the apparent potency of PI3Kδ inhibitors, their clinical use is hindered by side effects, including hepatitis, colitis, and pneumonitis. However, nanoparticle drug delivery systems have shown several merits in cancer treatment, such as achieving good pharmacokinetics, stability, and a reduction in side effects [44]. Preclinical research showed that PI3Kδ inhibitors’ side effects can be considerably reduced by the use of nanoparticle delivery systems. For example, Au et al. studied the delivery of BEZ235, which is a PI3Kδ inhibitor, by nanoparticles and showed a considerable reduction in its toxicity and side effects. The use of DFT calculations to evaluate the drug’s potential for nanoparticle delivery has gained considerable momentum in recent years [19]. This is because DFT calculations have the advantage of saving time and money compared to laboratory techniques used in nanoparticle formulation and development [45]. As an example, Coronilla et al. performed theoretical investigations by DFT calculations on the interactions between ibrutinib and gold nanoparticles to determine the possibility of being used as a drug delivery system in chronic lymphocytic leukaemia [21]. The present study attempted to determine the potential of gold nanoparticles as a drug delivery system for umbralisib analogue 306 using the DFT calculations regarding their electronic energies. Based on the DFT, it is found that the non-covalent interactions between analogue 306 and the gold nanoparticle were favourable and broken after the optimisation. This would ensure the effective delivery and release of analogue 306 at the site of action. Based on these findings, gold nanoparticles may be used as a delivery system for analogue 306 [46].

## 5. Conclusions

PI3Kδ inhibitors such as umbralisib play a significant role in the treatment of non-Hodgkin’s lymphoma. Thus, we utilised computer-aided drug design methods to design new umbralisib analogues. Based on the binding affinity and interaction analysis, eleven compounds were shortlisted. Further, based on MM-GBSA and ADMET analysis, among the shortlisted, analogue 306 was selected for MD simulation due to its promising results in percent human oral absorption and binding free energy. The MD simulation of analogue 306 and umbralisib in complex with PI3Kδ revealed that analogue 306 formed a more stable complex in the ligand-binding pocket of PI3Kδ during 100 ns simulation. DFT studies on analogue 306 have found stable interactions with gold through oxygen number 5. The new inhibitor showed promising in silico results and may become potential drug candidates or, at the very least, they may stimulate new strategies for developing novel inhibitors against PI3Kδ. However, the synthesis and substantiation of this compound through in vitro analysis remain necessary to confirm the potency of the anticancer activity shown by this compound.

## Figures and Tables

**Table 1:** Quantum polarised ligand docking of umbralisib with 4XE0.

Number	Docking Score Kcal/mol	XP Pose Rank	RMSD Å
Pose1	−7.49	1	0.39
Pose2	−7.46	1	0.37
Pose3	−7.25	1	0.31
Pose4	−7.10	2	0.30
Pose5	−7.08	2	0.29
Pose6	−7.05	3	0.31
Pose7	−7.02	2	0.34
Pose8	−6.92	1	0.28
Pose9	−6.80	3	0.37
Pose10	−6.74	2	0.30

**Table 2:** Docking and MM/GBSA results for top eleven analogues.

Title	Docking Score (Kcal/mol)	XP GScore (Kcal/mol)	MM-GBSA dG Bind (K/mol)	Number of Interaction Bonds (Kcal/mol)	Interacting Residues with Distances (Å)	RMSD (Å)
Analogue 306	−7.68	−7.92	−52.22	3Pi-Pi,2H bond	TRP760 (4.86, 5.34 5.25)-ASN836 (3.87)-SER831(2.29)	0.19
Analogue 268	−8.02	−8.02	−49.70	2Pi-Pi, 3H bonds	SER831(3.62)-VAL828 (3.78) ASN836 (3.81) TRP760 (4.89,5.28)	0.23
Analogue 205	−7.89	−7.90	−49.60	3Pi-Pi,2H bond	HIS830 (4.16)-VAL828 (2.77)-TRP760 (5.06, 3.97, 5.31)	1.35
Analogue 202	−7.92	−7.92	−49.54	3Pi-Pi,1H bond	ASN836 (4.15)-TRP760 (3.94, 4.13, 5.37)	0.43
Analogue 262	−8.42	−8.42	−48.51	3Pi-Pi,3H bond	VAL828 (3.79)-SER831 (4.08)-ASN836 (3.99)-TRP760 (5.36, 5.46, 5.06)	0.20
Analogue 101	−8.24	−8.24	−48.14	3Pi-Pi,1H bond	TRP760 (5.32, 4.49, 4.32)-ASN836 (4.14)	0.25
Analogue 131	−7.72	−7.72	−47.47	3Pi-Pi,1H bond	TRP760 (5.35, 4.95, 5.20)-ASN836 (2.97)	0.56
Analogue 223	−8.10	−8.16	−47.34	2Pi-Pi,1H bond	TRP760 (5.33)-His830 (2.23)-HIS830 (4.79)	0.55
Analogue 201	−7.83	−7.83	−46.75	3Pi-Pi,2H bond	TRP760 (5.29, 4.18, 4.33)-HIS830 (1.82)-ASN836 (4.09)	1.46
Analogue 293	−7.66	−7.66	−45.39	3Pi-Pi,1H bond	TRP760 (5.26, 4.87, 5.19)-ASN836(3.86)	0.15
Analogue 188	−7.81	−7.81	−44.59	1Pi-Pi,1H bond	TRY813 (4.48)-PHE751 (2.49)	0.55
Umbralisib	−7.58	−7.58	−44.44	3Pi-Pi,1 H bond	TRP760-ASN836	0.64

**Table 3:** Predicted ADMET properties of the top eleven designed analogues.

Compound	QPlog Po/w ^a^	QPlog S ^b^	QPlog HERG ^c^	QPPCaco ^d^	QPlogBB ^e^	HOA ^f^	HD ^g^	HA ^h^	MW ^i^	ROF ^j^
Analogue 306	7.35	−9.45	−7.61	631.52	−1.10	94.18	1	10	651.64	2
Analogue 268	6.67	−9.05	−7.23	349.30	−1.48	85.62	2	9	641.64	2
Analogue 205	5.73	−8.10	−6.55	317.40	−1.27	79.35	1	9	613.59	2
Analogue 202	6.13	−8.51	−6.68	537.57	−1.02	85.84	2	8	600.59	2
Analogue 262	4.59	−7.05	−5.87	39.45	−2.18	56.50	3	9	629.59	1
Analogue 101	5.81	−7.72	−6.58	550.31	−0.97	84.11	1	8	587.55	2
Analogue 131	5.46	−7.89	−6.62	241.92	−1.41	75.66	1	8	586.57	2
Analogue 223	6.19	−9.06	−7.63	337.04	−1.39	82.52	2	9	619.62	2
Analogue 201	5.67	−8.02	−4.82	30.47	−1.77	60.8	1	10	615.56	2
Analogue 293	6.031	−8.54	−7.24	182.75	−1.89	63.86	1	10	692.71	2
Analogue 188	7.06	−10.02	−7.93	616.33	−1.02	92.29	1	10	636.67	2
Umbralisib	6.50	−8.58	−6.84	822.55	−0.72	91.30	1	8	571.55	2

Notes: Predicted ^a^ octanol/water partition coefficient log P (acceptable range −2.0–6.5). Predicted ^b^ aqueous solubility in mol/L (acceptable range −6.5–0.5). Predicted ^c^ IC_50_ value for blockage of HERG K^+^ channels (concern below −5). Predicted ^d^ caco cell permeability in nm/s (<25 is poor <500 is great). Predicted ^e^ blood–brain barrier permeability (acceptable range −3–1.2). The percentage ^f^ of human oral absorption (the acceptable range more of than 80% is high and less than 25% is low. Number ^g^ of hydrogen bond donors. Number ^h^ of hydrogen bond acceptors. Molecular ^i^ weight. Lipinski ^j^ rule of 5.

**Table 4:** In silico predicted toxicity of the top compounds.

Compound	Oral Toxicity		Organ Toxicity	Toxicity Endpoints			
	Toxicity Class	LD50	Hepato Toxicity	Immuno Toxicity	Mutagenicity	Cyto Toxicity	Carcinogenicity
Analogue 306	IV	1000 mg/kg	+0.55	+0.90	−0.52	−0.61	−0.54
Analogue 268	IV	1000 mg/kg	+0.55	+0.97	−0.57	−0.61	−0.55
Analogue 205	IV	1000 mg/kg	+0.62	+0.96	+0.52	−0.68	−0.54
Analogue 202	IV	1000 mg/kg	+0.65	+0.96	+0.53	−0.60	−0.52
Analogue 262	IV	1000 mg/kg	+0.59	−0.82	−0.55	−0.72	−0.56
Analogue 101	IV	1000 mg/kg	+0.55	+0.97	−0.57	−0.61	−0.55
Analogue 131	IV	1000 mg/kg	+0.65	+0.93	−0.51	−0.64	−0.50
Analogue 223	IV	1000 mg/kg	+0.65	+0.87	+0.51	−0.64	−0.52
Analogue 201	IV	1000 mg/kg	+0.62	+0.95	+0.53	−0.69	−0.54
Analogue 293	IV	1000 mg/kg	+0.63	+0.89	−0.57	−0.64	+0.50
Analogue 188	IV	1000 mg/kg	+0.63	−0.51	+0.53	−0.66	−0.55
Umbralisib	IV	1000 mg/kg	+0.64	+0.74	+0.51	−0.64	−0.52

Note: LD50 (lethal dose in mg/kg), − (inactive toxic class with probability score), + (active toxic class with probability score).

**Table 5:** The gold interaction energies with oxygen and nitrogen atoms of analogue 306.

Complex	Interacting Atom	Complex EE + ZPE(Hartree)	Interaction Energy (Hartree)	Interaction Energy (Kcal/mol)
M1	Nitrogen 70	−2800.675858	−0.039173	−24.581
M2	Nitrogen 10	−2800.675672	−0.038987	−24.464
M3	Nitrogen 11	−2800.675687	−0.039002	−24.474
M4	Oxygen 6	−2800.671692	−0.035007	−21.967
M5	Nitrogen 9	−2800.675672	−0.03897	−24.454
M6	Nitrogen 7	−2800.675702	−0.039017	−24.483
M7	Nitrogen 8	−2800.675704	−0.039019	−24.484
M8	Oxygen 4	−2800.675678	−0.039	−24.472
M9	Nitrogen 67	−2900.675857	−0.039172	−24.580
M10	Oxygen 5	−2800.683565	−0.046887	−29.422

## Data Availability

The datasets generated during and/or analysed during the current study are available from the corresponding author upon reasonable request.

## Supplementary Materials

The following supporting information can be downloaded at: https://www.mdpi.com/article/10.3390/molecules28052289/s1, Figure S1. 2D interaction of the remaining top eight analogues in complex with PI3Kδ protein (PDB ID:4XE0) using the XP docking mode of Glide software. The hydrogen bond interactions with residues are represented by a purple dashed arrow directed towards the electron donor. The hydrophobic residues are in green color. Table S1: Docking scores of the designed analogues.
