# Supplementary material for: Design of Novel Phosphatidylinositol 3-Kinase Inhibitors for Non-Hodgkin’s Lymphoma: Molecular Docking, Molecular Dynamics, and Density Functional Theory Studies on Gold Nanoparticles
Source: Molecules. 2023 Mar 1;28(5):2289. doi: 10.3390/molecules28052289 (PMC10005307; doi:10.3390/molecules28052289)
Supplement: Supplementary file 1 [file molecules-28-02289-s001.zip › molecules-2235134-supplementary.pdf]

molecules

MDPI

Article

Design of Novel Phosphoinositol 3-kinase Inhibitors for Non-Hodgkin's Lymphoma: Molecular Docking, Molecular Dynamics, and Density Functional Theory Studies on Gold Nanoparticles

Abdalrahim M. Ali[1], Alaa A. Makki[1], Walaa Ibraheem[1], Mohammed Abdelrahman[2], Wadah Osman[3,4], Asmaa E. Sherif[3,5], Ahmed Ashour[3,5], Sabrin R. M. Ibrahim[6,7], Kholoud F. Ghazawi[8], Waad A. Samman[9], Abdulrahim A. Alzain[1]*

[1]Department of Pharmaceutical Chemistry, Faculty of Pharmacy, University of Gezira, Gezira, Sudan
[2]Department of Pharmaceutics, Faculty of Pharmacy, University of Gezira, Gezira, Sudan
[3]Department of Pharmacognosy, Faculty of Pharmacy, Prince Sattam Bin Abdulaziz University, Al-kharj 11942, Saudi Arabia
[4]Department of Pharmacognosy, Faculty of Pharmacy, University of Khartoum, Al-Qasr Ave, 11111, Khartoum, Sudan
[5]Department of Pharmacognosy, Faculty of Pharmacy, Mansoura University, Mansoura 35516, Egypt
[6] Preparatory Year Program, Department of Chemistry, Batterjee Medical College, Jeddah 21442, Saudi Arabia
[7] Department of Pharmacognosy, Faculty of Pharmacy, Assiut University, Assiut 71526, Egypt
[8] Clinical Pharmacy Department, College of Pharmacy, Umm Al-Qura University, Makkah 24382, Saudi Arabia
[9] Department of Pharmacology and Toxicology, College of Pharmacy, Taibah University, Al-Madinah Al-Munawwarah 30078, Saudi Arabia

*Corresponding author: Abdulrahim A. Alzain,
E-mail: abdulrahim.altoam@uofg.edu.sd; abdulrahim.altoam@gmail.com (A.A.A)

molecules
MDPI

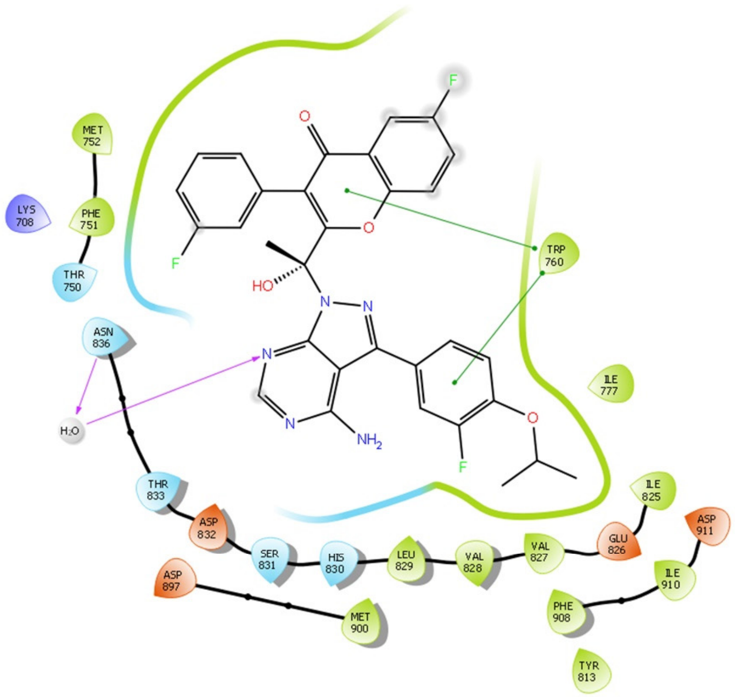

analogue 101

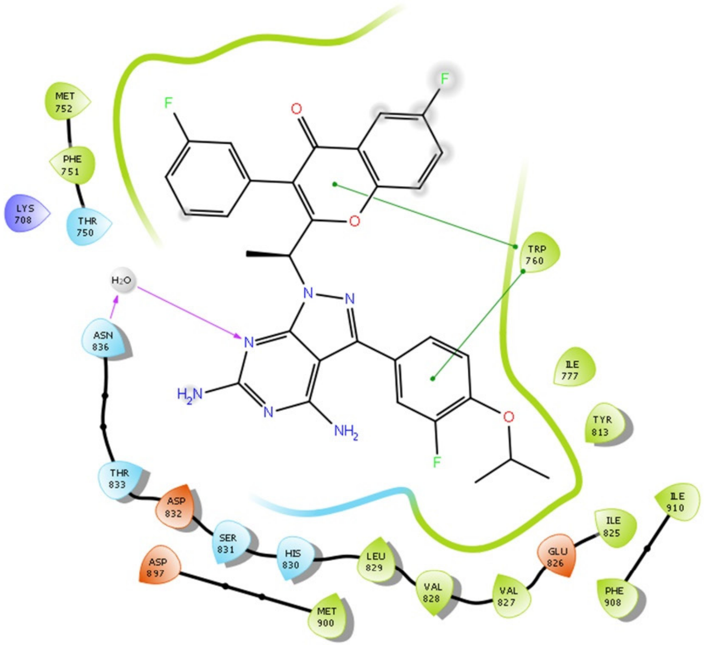

analogue 131

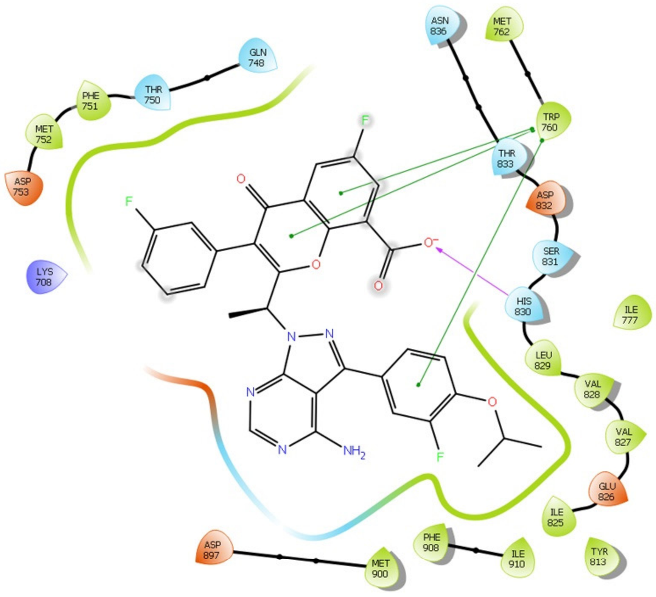

analogue 201

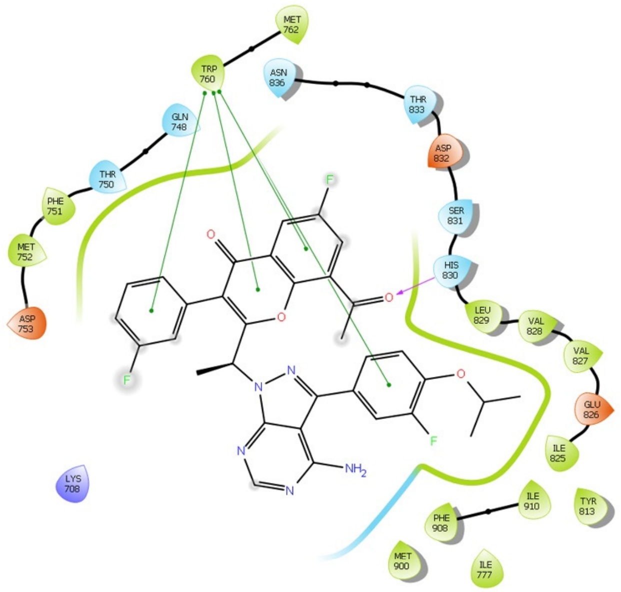

analogue 205

molecules MDPI

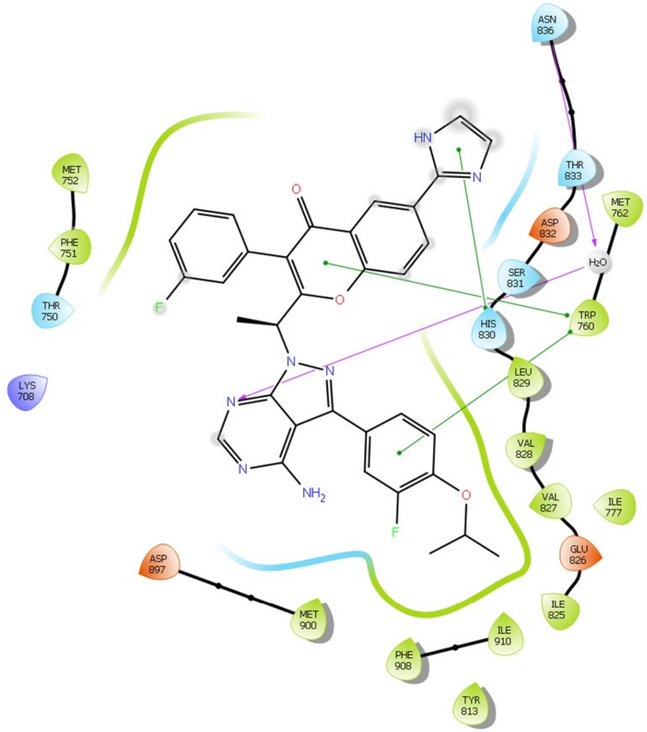

analogue 223

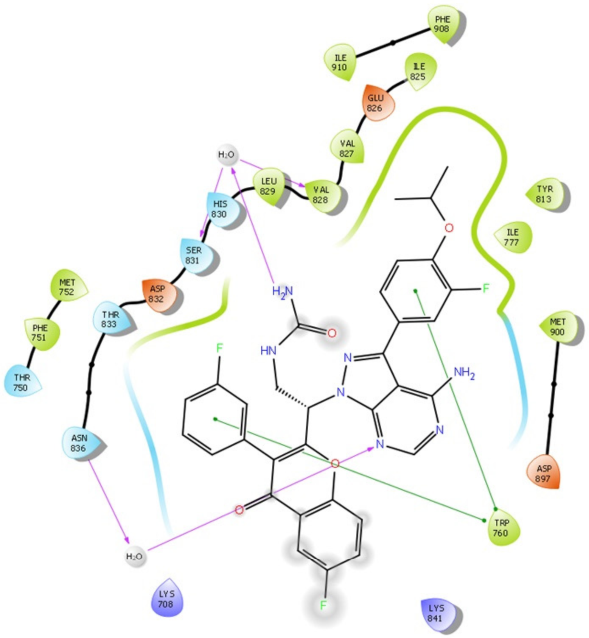

analogue 262

molecules

MDPI

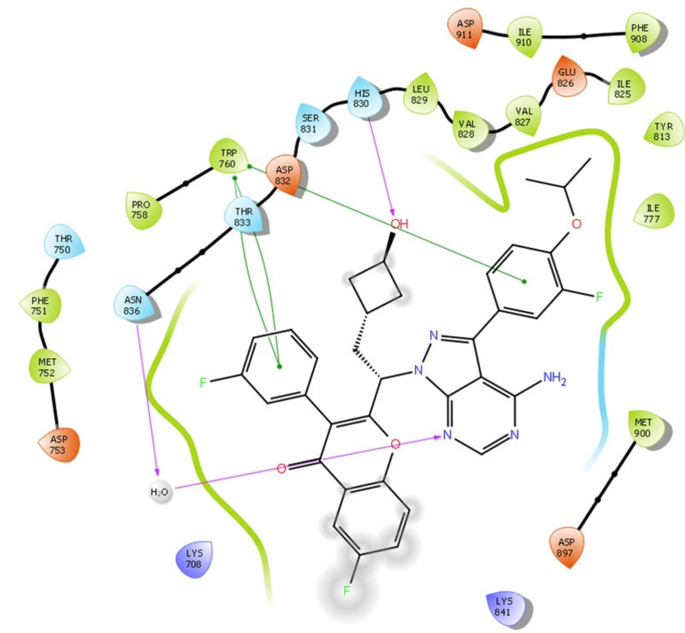

analogue 268

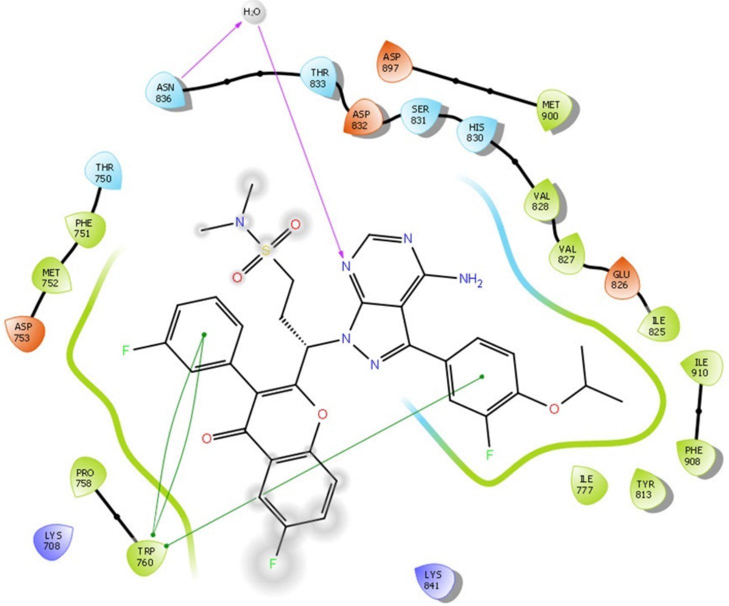

analogue 293

Figure S1. 2D interaction of the remaining top eight analogues in complex with PI3Kδ protein (PDB ID:4XE0) using the XP docking mode of Glide software. The hydrogen bond interactions with residues are represented by a purple dashed arrow directed towards the electron donor. The hydrophobic residues are in green color.

molecules

MDPI

Table S1: Docking scores of the designed analogues.

Title	Structure	docking score<br><br>(kxal/mol)
Analoguue1	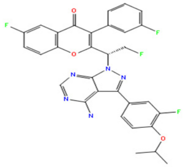	-7.262
Analoguue2	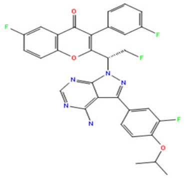	-7.228
Analoguue3	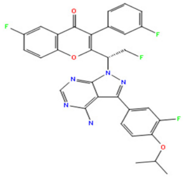	-7.226

Analoguue4	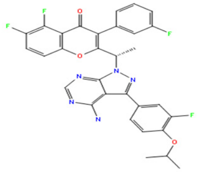	-7.286
Analoguue5	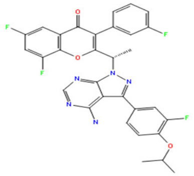	-7.684
Analoguue6	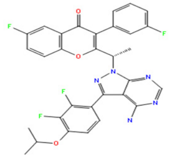	-7.291
Analoguue7	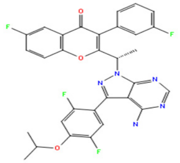	-7.017

molecules MDPI

Analoguue8	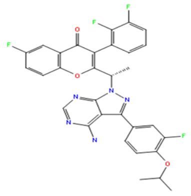	-7.302
Analoguue9	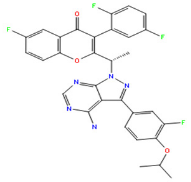	-7.110
Analoguue10	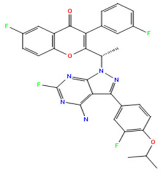	-6.884
Analoguue11	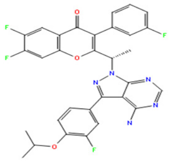	-7.169

molecules

MDPI

Analoguue12	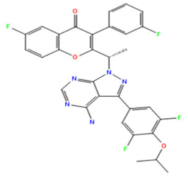	-7.656
Analoguue13	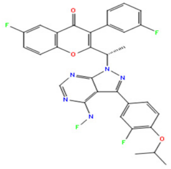	-7.243
Analoguue14	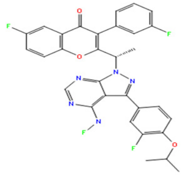	-7.081
Analoguue15	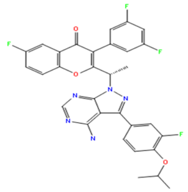	-7.148

Analoguue16	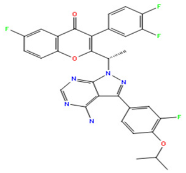	-7.575
Analoguue17	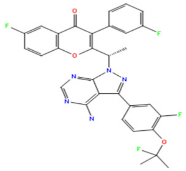	-7.097
Analoguue18	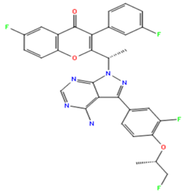	-6.178
Analoguue19	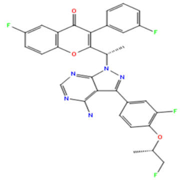	-7.280

Analoguue20	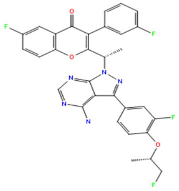	-7.207
Analoguue21	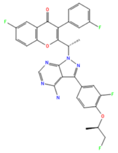	-7.239
Analoguue22	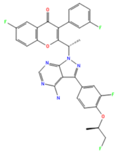	-7.555
Analoguue23	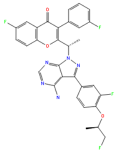	-7.569

Analoguue24	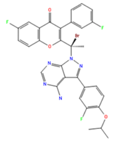	-7.547
Analoguue25	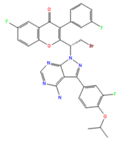	-7.400
Analoguue26	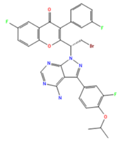	-7.467
Analoguue27	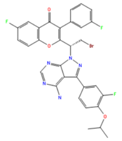	-7.450

molecules
MDPI

Analoguue28	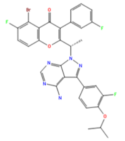	-7.480
Analoguue29	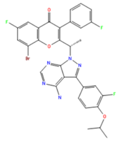	-7.731
Analoguue30	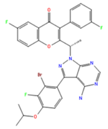	-7.269
Analoguue31	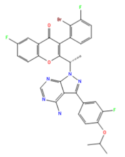	10000.000

Analoguue32	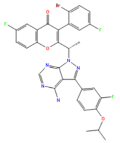	-7.487
Analoguue33	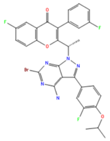	10000.000
Analoguue34	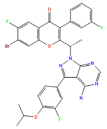	-7.350
Analoguue35	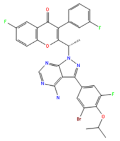	-7.676

molecules MDPI

Analoguue36	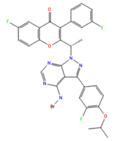	10000.000
Analoguue37	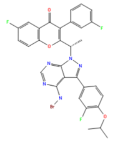	-7.566
Analoguue38	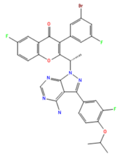	-6.797
Analoguue39	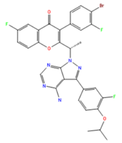	-7.170

Analoguue40	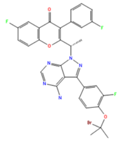	10000.000
Analoguue41	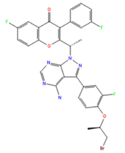	10000.000
Analoguue42	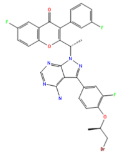	-6.639
Analoguue43	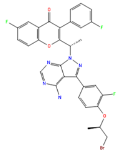	-6.479

Analoguue44	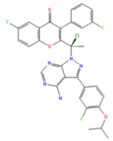	-6.423
Analoguue45	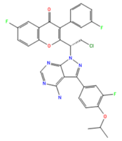	-7.364
Analoguue46	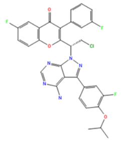	-7.332
Analoguue47	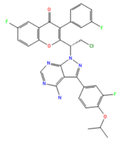	-7.329

molecules
MDPI

Analoguue48	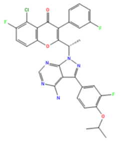	-7.337
Analoguue49	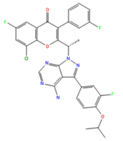	-7.603
Analoguue50	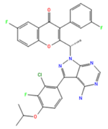	-7.278
Analoguue51	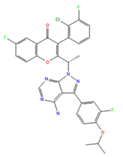	10000.000

Analoguue52	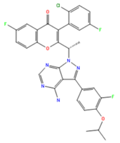	-7.398
Analoguue53	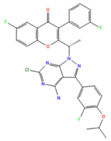	-5.203
Analoguue54	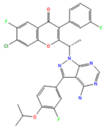	-7.271
Analoguue55	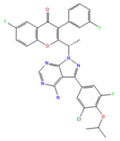	-7.574

Analoguue56	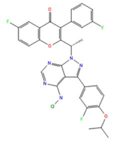	-5.336
Analoguue57	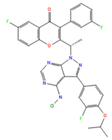	-6.924
Analoguue58	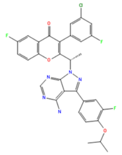	-6.729
Analoguue59	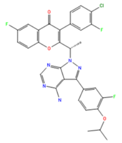	-7.252

Analoguue60	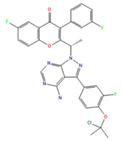	10000.000
Analoguue61	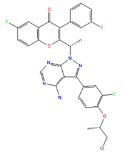	-5.516
Analoguue62	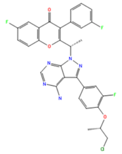	10000.000
Analoguue63	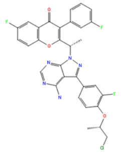	-4.993

molecules

MDPI

Analoguue64	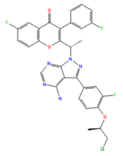	-5.111
Analoguue65	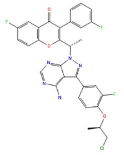	-7.069
Analoguue66	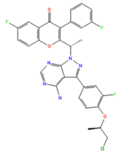	-7.038
Analoguue67	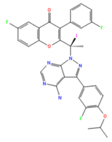	-6.990

molecules MDPI

Analoguue68	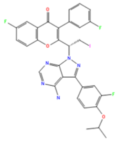	-7.577
Analoguue69	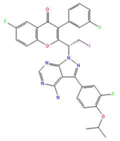	-7.539
Analoguue70	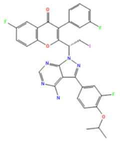	-7.535
Analoguue71	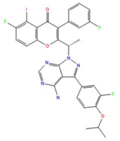	-7.544

Analoguue72	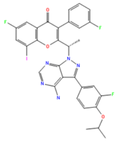	-7.791
Analoguue73	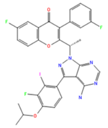	-7.428
Analoguue74	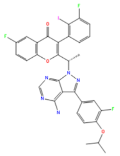	10000.000
Analoguue75	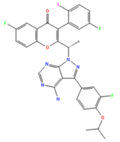	-7.520

Analoguue76	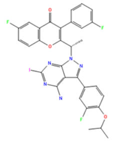	-5.730
Analoguue77	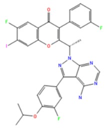	-7.485
Analoguue78	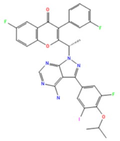	-7.663
Analoguue79	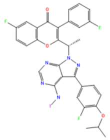	-5.949

Analoguue80	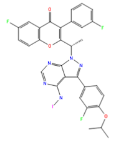	-7.642
Analoguue81	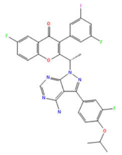	-7.522
Analoguue82	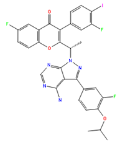	-7.485
Analoguue83	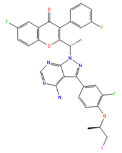	10000.000

Analoguue84	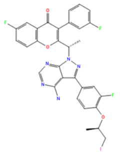	-6.884
Analoguue85	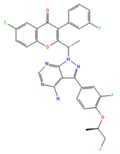	-6.906
Analoguue86	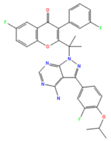	-6.924
Analoguue87	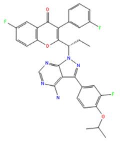	-7.362

molecules MDPI

Analoguue88	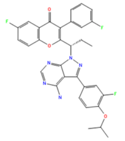	-7.233
Analoguue89	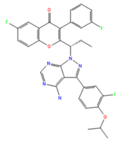	-7.215
Analoguue90	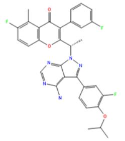	-7.210
Analoguue91	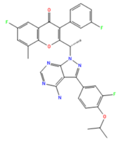	-7.507

Analoguue92	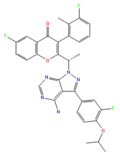	-7.017
Analoguue93	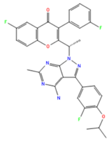	-7.310
Analoguue94	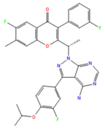	-7.417
Analoguue95	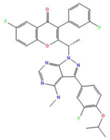	-7.307

Analoguue96	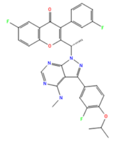	-7.268
Analoguue97	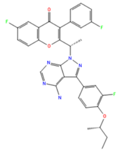	-7.314
Analoguue98	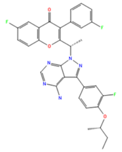	-6.096
Analoguue99	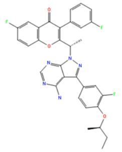	-5.259

Analoguue100	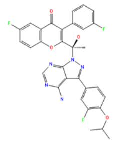	-6.725
Analoguue101	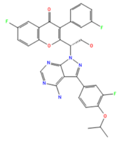	-8.242
Analoguue102	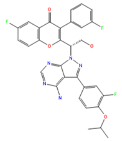	-7.438
Analoguue103	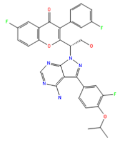	-7.443

molecules

Analoguue104	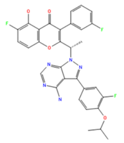	-7.236
Analoguue105	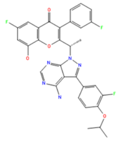	-7.344
Analoguue106	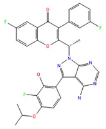	-7.426
Analoguue107	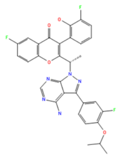	-6.232

Analoguue108	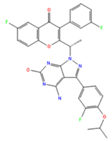	-7.410
Analoguue109	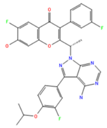	-7.800
Analoguue110	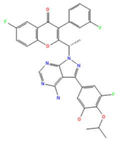	-7.469
Analoguue111	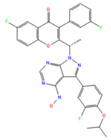	-6.456

molecules MDPI

Analoguue112	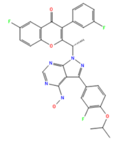	-7.372
Analoguue113	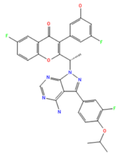	-7.365
Analoguue114	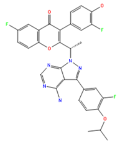	-7.277
Analoguue115	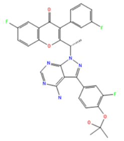	-7.281

molecules

Analoguue116	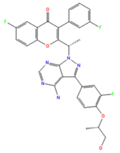	-6.680
Analoguue117	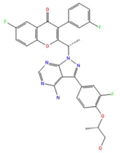	-6.556
Analoguue118	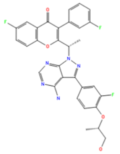	-6.372
Analoguue119	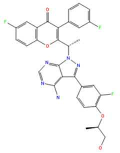	-6.357

Analoguue120	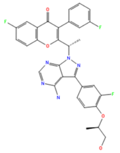	-7.673
Analoguue121	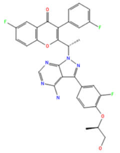	-7.665
Analoguue122	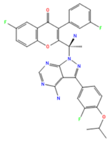	-7.484
Analoguue123	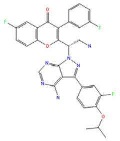	-7.376

molecules

MDPI

Analoguue124	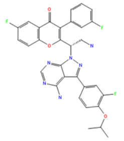	-7.179
Analoguue125	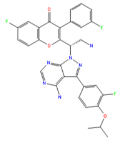	-7.177
Analoguue126	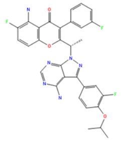	-7.177
Analoguue127	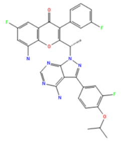	-7.386

molecules
MDPI

Analoguue128	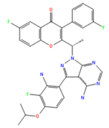	-7.372
Analoguue129	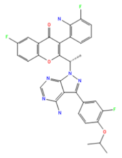	-6.069
Analoguue130	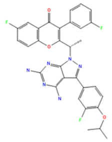	-7.324
Analoguue131	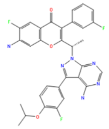	-7.721

molecules MDPI

Analoguue132	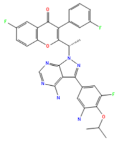	-7.399
Analoguue133	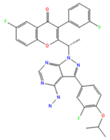	-5.842
Analoguue134	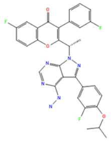	-7.288
Analoguue135	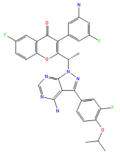	-7.281

molecules MDPI

Analoguue136	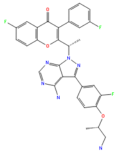	-6.939
Analoguue137	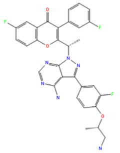	10000.000
Analoguue138	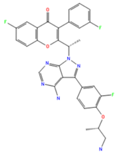	10000.000
Analoguue139	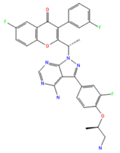	10000.000

molecules MDPI

Analoguue140	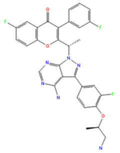	-6.899
Analoguue141	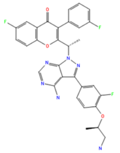	-7.140
Analoguue142	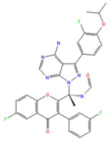	-6.964
Analoguue143	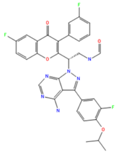	-7.542

Analoguue144	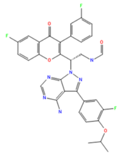	-7.208
Analoguue145	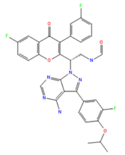	-7.202
Analoguue146	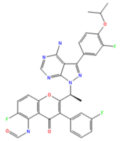	-7.202
Analoguue147	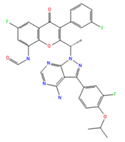	-7.633

Analoguue148	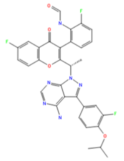	-8.119
Analoguue149	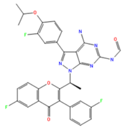	-7.397
Analoguue150	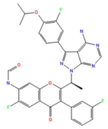	-7.703
Analoguue151	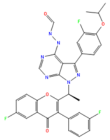	-7.514

Analoguue152	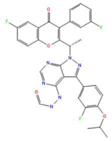	-7.444
Analoguue153	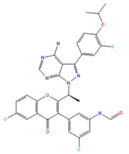	-7.717
Analoguue154	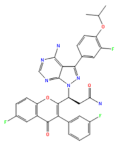	-6.188
Analoguue155	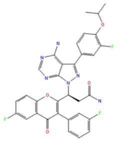	-7.028

molecules MDPI

Analoguue156	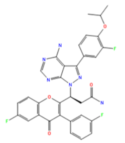	-7.209
Analoguue157	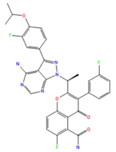	-7.054
Analoguue158	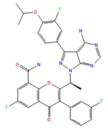	-7.505
Analoguue159	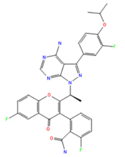	-7.711

molecules

MDPI

Analoguue160	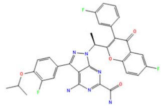	-7.580
Analoguue161	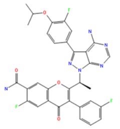	-7.499
Analoguue162	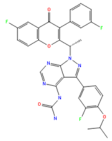	-7.922
Analoguue163	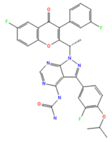	-6.344

Analoguue164	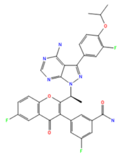	-4.694
Analoguue165	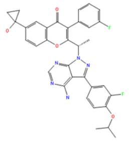	-7.041
Analoguue166	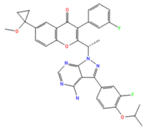	-7.636
Analoguue167	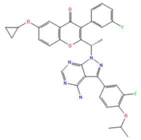	-7.434

Analoguue168	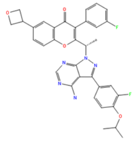	-7.635
Analoguue169	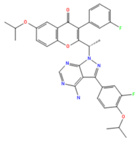	-7.549
Analoguue170	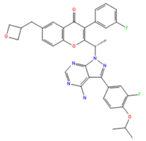	-7.296
Analoguue171	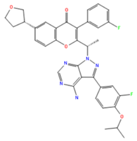	-7.920

Analoguue172	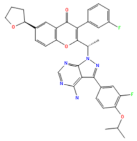	-7.697
Analoguue173	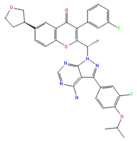	-7.833
Analoguue174	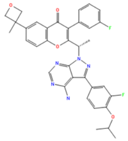	-8.461
Analoguue175	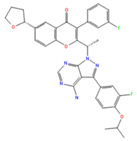	-7.779

Analoguue176	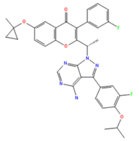	-7.734
Analoguue177	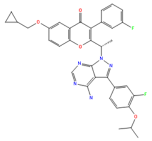	-7.923
Analoguue178	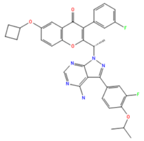	-7.497
Analoguue179	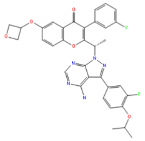	-7.822

molecules MDPI

Analoguue180	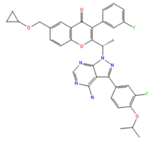	-7.546
Analoguue181	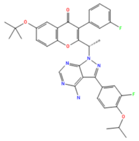	-7.588
Analoguue182	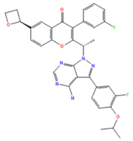	-7.785
Analoguue183	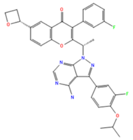	-7.672

molecules MDPI

Analoguue184	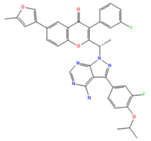	-7.444
Analoguue185	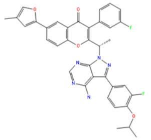	-7.542
Analoguue186	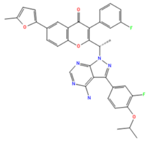	-7.791
Analoguue187	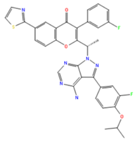	-7.657

molecules MDPI

Analoguue188	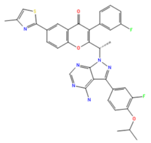	-7.816
Analoguue189	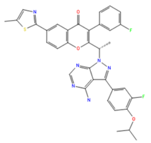	-7.900
Analoguue190	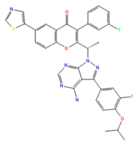	-7.797
Analoguue191	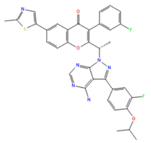	-7.904

molecules MDPI

Analoguue192	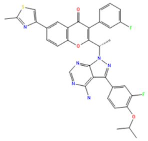	-7.756
Analoguue193	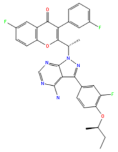	-7.704
Analoguue194	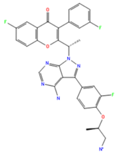	-5.903
Analoguue195	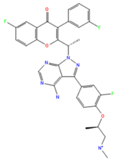	-6.822

Analoguue196	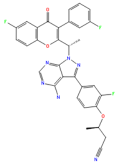	10000.000
Analoguue197	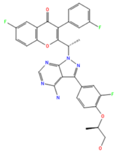	10000.000
Analoguue198	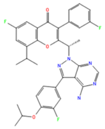	-7.683
Analoguue199	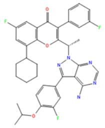	-7.135

Analoguue200	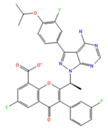	-7.123
Analoguue201	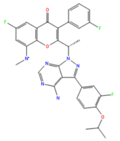	-7.834
Analoguue202	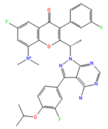	-7.923
Analoguue203	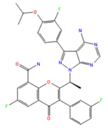	-7.398

molecules

MDPI

Analoguue204	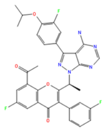	-6.573
Analoguue205	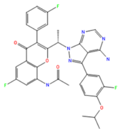	-7.894
Analoguue206	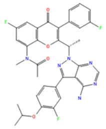	-7.908
Analoguue207	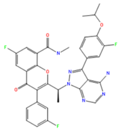	-4.389

molecules MDPI

Analoguue208	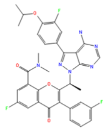	-3.753
Analoguue209	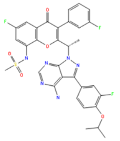	-5.882
Analoguue210	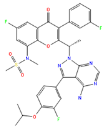	-8.298
Analoguue211	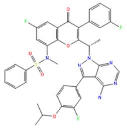	-6.754

molecules MDPI

Analoguue212	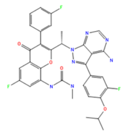	-4.672
Analoguue213	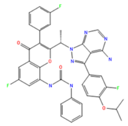	-8.155
Analoguue214	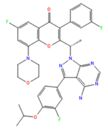	-6.735
Analoguue215	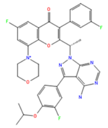	-6.789

Analoguue216	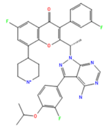	-7.261
Analoguue217	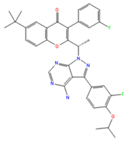	-7.576
Analoguue218	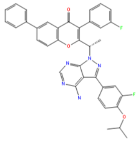	-7.567
Analoguue219	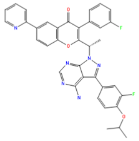	-7.828

Analoguue220	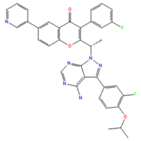	-7.702
Analoguue221	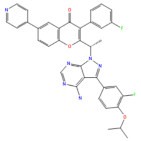	-7.806
Analoguue222	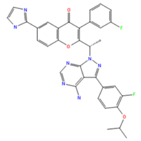	-7.725
Analoguue223	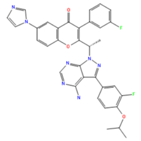	-8.103

Analoguue224	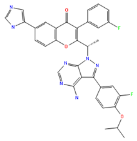	-7.633
Analoguue225	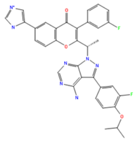	-7.531
Analoguue226	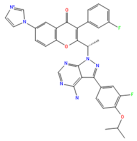	-7.820
Analoguue227	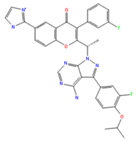	-7.958

molecules MDPI

Analoguue228	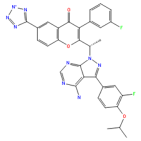	-7.787
Analoguue229	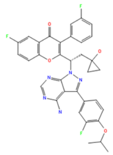	-7.357
Analoguue230	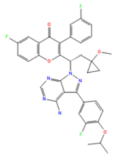	-7.257
Analoguue231	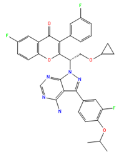	-7.348

molecules MDPI

Analoguue232	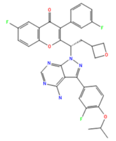	-7.377
Analoguue233	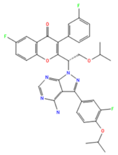	-7.289
Analoguue234	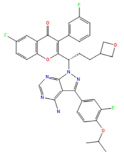	-7.391
Analoguue235	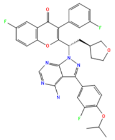	-7.350

Analoguue236	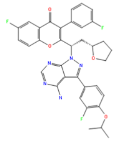	-7.371
Analoguue237	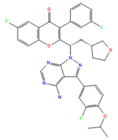	-7.298
Analoguue238	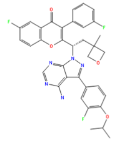	-7.395
Analoguue239	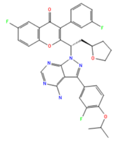	-7.397

Analoguue240	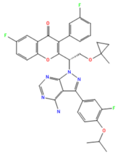	-7.297
Analoguue241	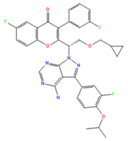	-7.412
Analoguue242	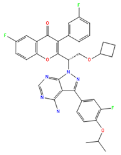	-7.383
Analoguue243	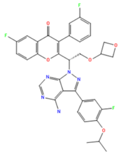	-7.418

molecules MDPI

Analoguue244	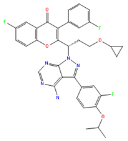	-7.502
Analoguue245	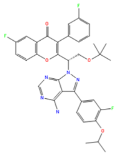	-7.337
Analoguue246	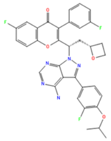	-7.237
Analoguue247	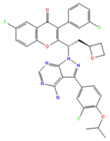	-7.184

Analoguue248	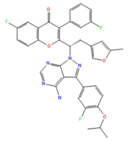	-7.343
Analoguue249	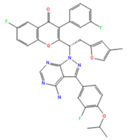	-7.467
Analoguue250	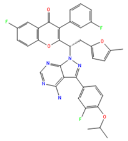	-7.462
Analoguue251	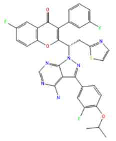	-7.395

Analoguue252	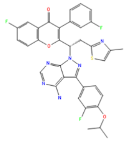	-7.356
Analoguue253	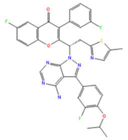	-7.366
Analoguue254	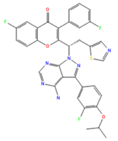	-7.370
Analoguue255	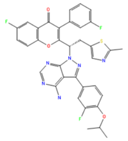	-7.362

Analoguue256	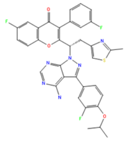	-7.371
Analoguue257	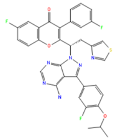	-7.415
Analoguue258	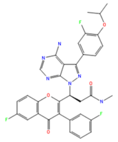	-7.346
Analoguue259	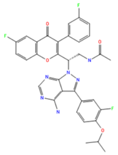	-7.479

molecules MDPI

Analoguue260	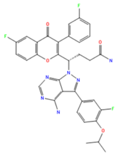	-7.219
Analoguue261	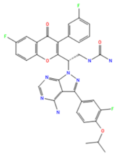	-7.683
Analoguue262	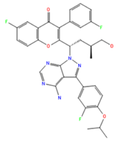	-8.425
Analoguue263	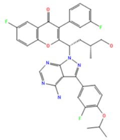	-7.159

Analoguue264	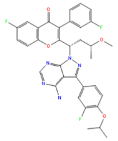	-7.365
Analoguue265	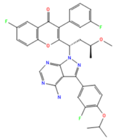	-7.378
Analoguue266	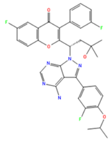	-7.265
Analoguue267	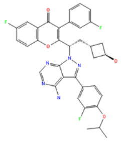	-7.295

molecules MDPI

Analoguue268	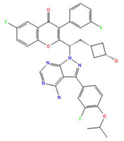	-8.023
Analoguue269	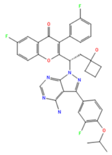	-7.553
Analoguue270	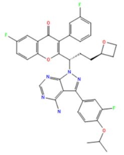	-7.309
Analoguue271	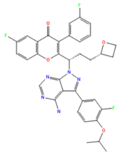	-7.376

Analoguue272	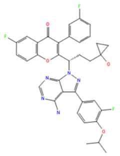	-7.370
Analoguue273	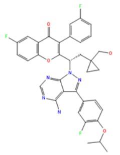	-7.446
Analoguue274	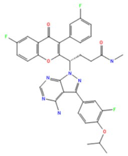	-7.487
Analoguue275	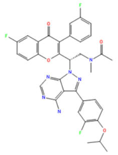	-7.397

molecules MDPI

Analoguue276	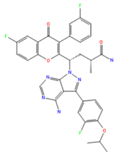	-7.297
Analoguue277	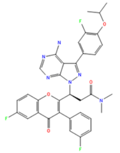	-7.457
Analoguue278	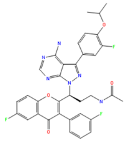	-7.117
Analoguue279	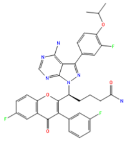	-7.548

molecules MDPI

Analoguue280	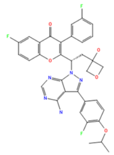	-7.116
Analoguue281	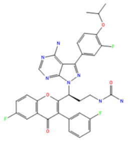	-7.284
Analoguue282	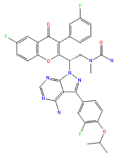	-7.873
Analoguue283	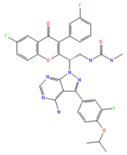	-7.330

molecules MDPI

Analoguue284	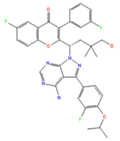	-7.518
Analoguue285	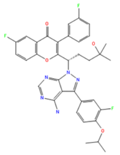	-7.307
Analoguue286	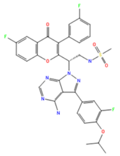	-7.637
Analoguue287	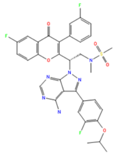	-7.527

molecules MDPI

Analoguue288	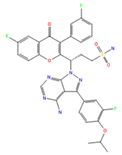	-7.370
Analoguue289	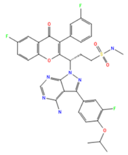	-7.478
Analoguue290	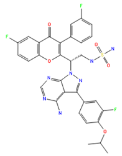	-7.709
Analoguue291	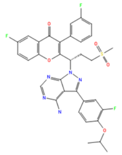	-7.404

molecules MDPI

Analoguue292	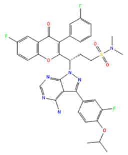	-7.242
Analoguue293	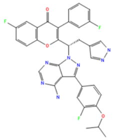	-7.667
Analoguue294	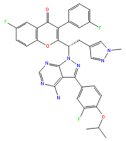	-7.639
Analoguue295	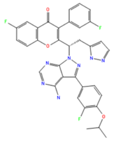	-7.636

molecules MDPI

Analoguue296	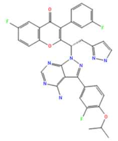	-7.267
Analoguue297	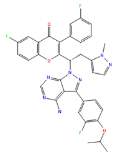	-7.284
Analoguue298	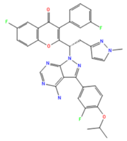	-7.404
Analoguue299	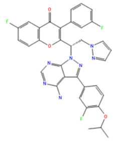	-7.350

molecules MDPI

Analoguue300	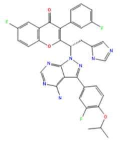	-7.280
Analoguue301	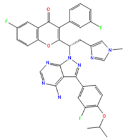	-7.586
Analoguue302	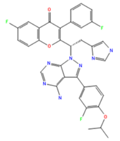	-7.585
Analoguue303	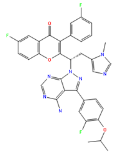	-7.311

molecules

Analoguue304	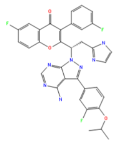	-7.419
Analoguue305	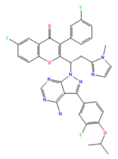	-7.317
Analoguue306	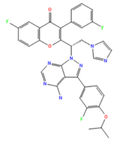	-7.682
Analoguue307	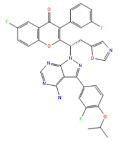	-7.275

Analoguue308	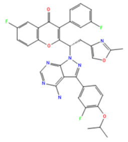	-7.202
Analoguue309	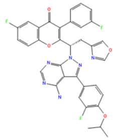	-7.316
Analoguue310	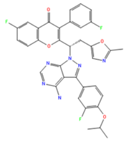	-7.306
Analoguue311	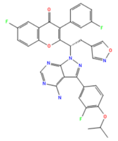	-7.330

Analoguue312	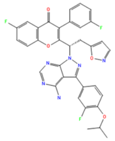	-7.341
Analoguue313	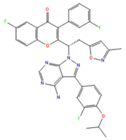	-7.451
Analoguue314	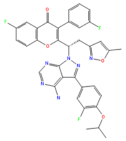	-7.525
Analoguue315	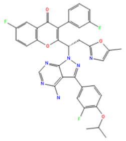	-7.516

molecules MDPI

Analoguue316	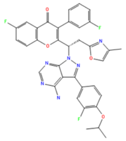	-7.362
Analoguue317	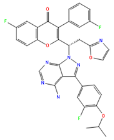	-7.260
Analoguue318	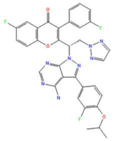	-7.191
Analoguue319	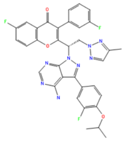	-7.232

Analoguue320	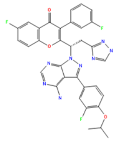	-7.370
Analoguue321	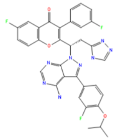	-7.230
Analoguue322	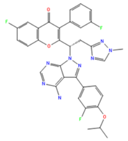	-7.646
Analoguue323	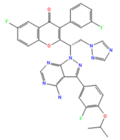	-7.295

Analoguue324	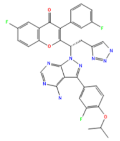	-7.191
Analoguue325	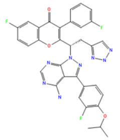	-7.292
Analoguue326	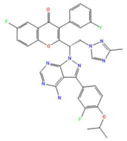	-7.349
Analoguue327	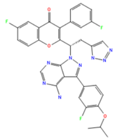	-7.436

molecules MDPI

Analoguue328	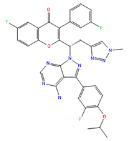	-7.362
Analoguue329	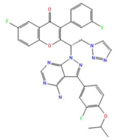	-7.649
Analoguue330	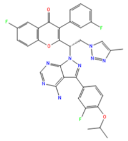	-7.272
Analoguue331	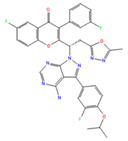	-7.303

molecules

MDPI

Analoguue332	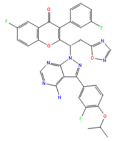	-7.245
Analoguue333	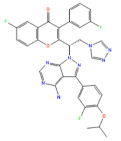	-7.200
Analoguue334	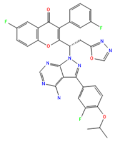	-7.322
Analoguue335	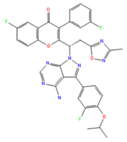	-7.274

molecules MDPI

Analoguue336	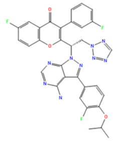	-7.245
Analoguue337	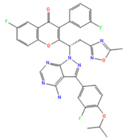	-7.234
Analoguue338	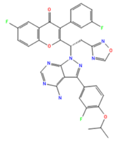	-7.254
Analoguue339	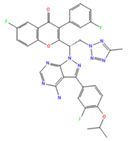	-7.225

molecules MDPI

Analoguue340	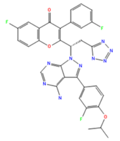	-7.266
Analoguue341	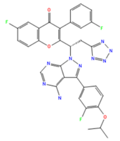	-7.299
Analoguue342	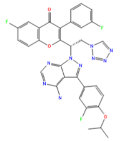	-7.258
Analoguue343	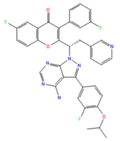	-7.314

molecules

MDPI

Analoguue344	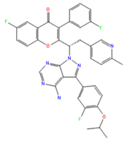	-7.445
Analoguue345	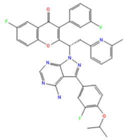	-7.415
Analoguue346	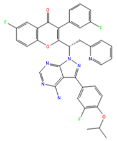	-7.344
Analoguue347	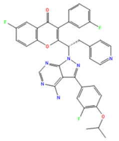	-7.331

Analoguue348	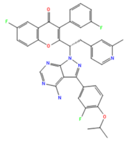	-7.368
Analoguue349	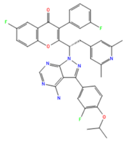	-7.411
Analoguue350	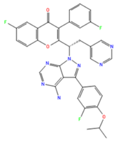	-7.423
Analoguue351	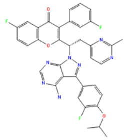	-7.352

molecules MDPI

Analoguue352	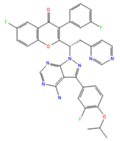	-7.459
Analoguue353	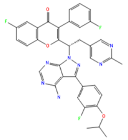	-7.322
Analoguue354	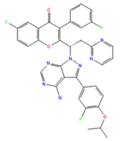	-7.404
Analoguue355	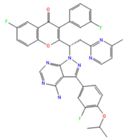	-7.357

Analoguue356	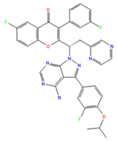	-7.420
Analoguue357	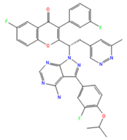	-7.335
Analoguue358	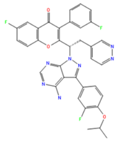	-7.488
Analoguue359	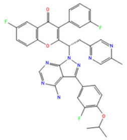	-7.452

molecules MDPI

Analoguue360	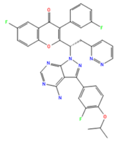	-7.364
Analoguue361	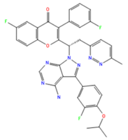	-7.254
Analoguue362	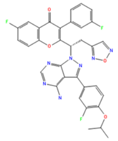	-7.426
Analoguue363	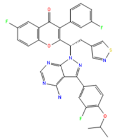	-7.280

molecules MDPI

Analoguue364	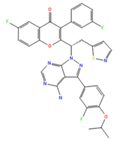	-7.505
Analoguue365	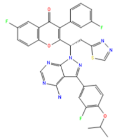	-7.344
Analoguue366	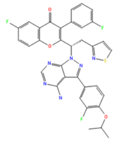	-7.342
Analoguue367	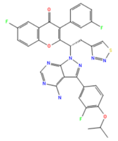	-7.566

molecules MDPI

Analoguue368	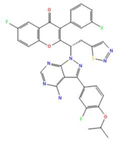	-7.378
Analoguue369	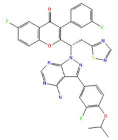	-7.378
Analoguue370	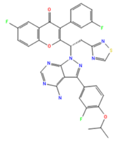	-7.353
Analoguue371	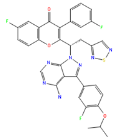	-7.241

Analoguue372	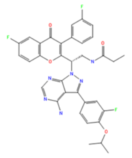	-7.333
Analoguue373	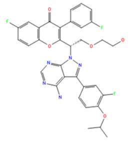	-7.219
Analoguue374	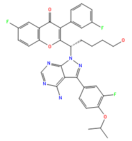	-7.693
Analoguue375	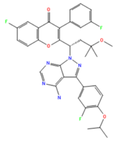	-7.585

Analoguue376	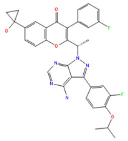	-7.353
Analoguue377	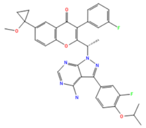	-7.636
Analoguue378	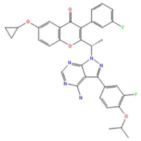	-7.434
Analoguue379	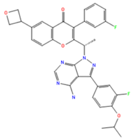	-7.635

molecules MDPI

Analoguue380	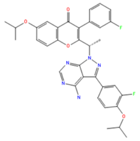	-7.549
Analoguue381	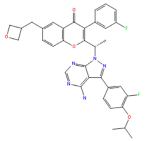	-7.296
Analoguue382	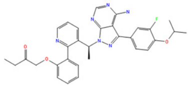	-7.920
Analoguue383	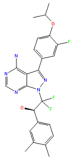	-6.151

molecules

MDPI

Analoguue384	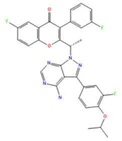	-5.023
Analoguue385	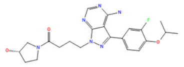	-5.845
Analoguue386	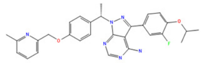	-5.601
Analoguue387	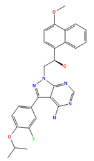	-5.735

molecules

MDPI

Analoguue388	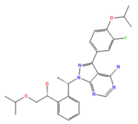	-4.764
Analoguue389	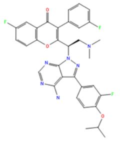	-5.307
Analoguue390	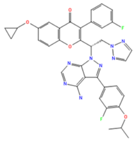	-7.092
Analoguue391	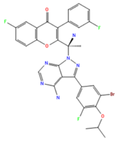	-6.727
